# SCF^SKP2^ regulates APC/C^CDH1^-mediated degradation of CTIP to adjust DNA-end resection in G_2_-phase

**DOI:** 10.1038/s41419-020-02755-9

**Published:** 2020-07-18

**Authors:** Fanghua Li, Emil Mladenov, Sharif Mortoga, George Iliakis

**Affiliations:** Institute of Medical Radiation Biology, University of Duisburg-Essen Medical School, 45122 Essen, Germany

**Keywords:** Post-translational modifications, Double-strand DNA breaks

## Abstract

The cell cycle-dependent engagement of DNA-end resection at DSBs is regulated by phosphorylation of CTIP by CDKs, the central regulators of cell cycle transitions. Cell cycle transitions are also intimately regulated by protein degradation via two E3 ubiquitin ligases: SCF^SKP2^ and APC/C^CDH1^ complex. Although APC/C^CDH1^ regulates CTIP in G_1_– and G_2_-phase, contributions by SCF^SKP2^ have not been reported. We demonstrate that SCF^SKP2^ is a strong positive regulator of resection. Knockdown of SKP2, fully suppresses resection in several cell lines. Notably, this suppression is G_2_-phase specific and is not observed in S-phase or G_1_–phase cells. Knockdown of SKP2 inactivates SCF^SKP2^ causing APC/C^CDH1^ activation, which degrades CTIP. The stabilizing function of SCF^SKP2^ on CTIP promotes resection and supports gene conversion (GC), alternative end joining (alt-EJ) and cell survival. We propose that CDKs and SCF^SKP2^-APC/C^CDH1^ cooperate to regulate resection and repair pathway choice at DSBs in G_2_-phase.

## Introduction

In higher eukaryotes, DSBs are processed by classical non-homologous end-joining (c-NHEJ) and gene-conversion (GC), while alternative end-joining (alt-EJ) and single-strand annealing (SSA) exert variable, context-dependent contributions^1–3^. c-NHEJ rejoins DNA ends after minimal processing without homology requirements. GC, SSA, and alt-EJ, process DNA ends to generate a 3′ single-stranded overhang, in a reaction termed DNA-end-resection, or simply resection^3–6^. GC and SSA require extensive homology, which for GC is found in the sister chromatid and in SSA in homologous regions in the vicinity of the DSB^7,8^. Short homologies are also utilized in alt-EJ^3^. Notably, only GC is conceptually designed to fully restore the genome and utilization of other pathways risks mutations and translocation-formation^9,10^ causing cell death or cancer^1^. Pathway choice is therefore a significant decision for the genetic stability of a damaged cell.

Resection is important in pathway choice because it suppresses c-NHEJ and clears the way for resection-dependent processing^3,4^. In the regulation of this decision, the cell cycle plays a central role at two levels. First, it progressively generates during S-phase the sister chromatid^11^. Second, it tightly controls the activities of several resection proteins, keeping them low in G_1_ and mediating a progressive increase in S- and G_2_-phase. Consequently, resection-dependent pathways are mainly active during S- and G_2_-phase, whereas c-NHEJ remains active throughout the cell cycle^12,13^.

It is now recognized that the resection apparatus is profoundly regulated by the cell cycle machinery, built around the cyclin-dependent Ser/Thr-kinases (CDKs)^3,4,11^. In mammals, cell cycle transitions are triggered by CDK4/6, CDK2, and CDK1, with overall activity low in G_1_ but rising progressively towards mitosis, enhancing in parallel resection^14^. Resection requires CTIP^15^ to stimulate MRE11 and proceeds bi-directionally^16,17^, with MRN proceeding in 3′–5′ direction, and EXO1 or BLM/DNA2 catalyzing long-range 5′–3′-resection^18^. CTIP phosphorylation by CDK on Thr847/Ser327 critically regulates resection^19–21^, while CDK2-dependent phosphorylation promotes CTIP binding to PIN1 to dampen resection^22^. In G_1_, phosphorylation of CTIP by PLK3 promotes limited resection^23^. CDK activity also promotes resection by phosphorylating EXO1^24^, NBS1^25–27^ and DNA2^28^. Finally, CDK activity promotes resection by suppressing resection-blocks raised by 53BP1 and HELB^29–31^, while cyclin D1 binds RAD51 to promote its recruitment to DSBs^32^.

Notably, the oscillating activity of CDKs is regulated by the periodic degradation of cyclins and CDK inhibitors (CKIs) by the ubiquitin–proteasome system to impose unidirectionality in cell cycle progression^33,34^. Central in this process is a pair of RING-type E3 ubiquitin ligases: SCF (SKP1/Cullin/F-box protein) and anaphase-promoting-complex/cyclosome (APC/C), that target proteins for proteasomal degradation using different strategies^35–37^. While both ligases retain low levels of activity throughout the cell cycle, SCF remains active from late-G_1_- to late-G_2_-phase and selectively degrades proteins primed for degradation—often by phosphorylation generating a specifically recognized phospho-degron. The S-phase kinase-associated protein 2 (SKP2) is the main substrate recognition factor of SCF, but alternative F-box protein partners, including β−TrCP, FBW7, and Cyclin F provide important functions^36^.

APC/C in contrast, is active only from late G_2_ to early G_1_ and catalyzes the destruction of entire populations of target proteins without requiring a specific posttranslational modification^33^. APC/C is present in two forms with partly overlapping substrate specificity: the first utilizes as targeting component CDC20 (APC/C^CDC20^) and is activated in late-G_2_- to early M-phase. The second utilizes as targeting component CDH1 (APC/C^CDH1^) and is activated in late M- to early/mid-G_1_-phase. CDH1 expression remains constant throughout the cell cycle, but its activity towards APC/C is suppressed during S/G_2_ by Cdk-phosphorylation that inhibits binding to the APC/C complex. Dephosphorylation of CDH1 by the CDC14B phosphatase allows binding to and activation of the APC/C complex late in G_2_-phase^38^. There is strong regulatory crosstalk between SCF and APC/C with active SCF suppressing the activity of APC/C^39^ and active APC/C suppressing the activity of SCF^36^. It is relevant that APC/C has ties to the resection apparatus^40^ with CTIP a key target^41^.

Despite intriguing connections between APC/C^CDH1^ and resection at DSBs, similar connections with SCF^SKP2^ have not been reported. This is surprising, because most connections reported for APC/C relate to functions in G_1_-phase. Here we show that SCF^SKP2^ positively regulates resection, specifically in G_2_-phase cells by suppressing APC/C^CDH1^ mediated degradation of CTIP. These results complete the link between resection and the cell cycle engine by adding the SCF^SKP2^-APC/C^CDH1^ protein degradation module to the CDKs.

## Materials and methods

### Cell culture and irradiation

Cells were grown at 37 °C in a humidified atmosphere of 5% CO_2_ in air. 82-6-hTert, AT hTert and HFF hTert fibroblasts were incubated in MEM cell culture medium, supplemented 10% fetal bovine serum (FBS) and 1% non-essential amino acids (NEA). A549 and U2OS were incubated in McCoy’s 5A medium, supplemented 10% FBS. HEK293 and RPE-1 were incubated in DMEM, supplemented 10% FBS (See supplementary methods for additional details.). All the cell lines used in the study were routinely tested for mycoplasma contamination, and only mycoplasma free cells were used in experiments. Cells were exposed to IR at room temperature (RT), unless mentioned specifically otherwise, using a 320 kV X-ray machine with a 1.65 Al filter (GE Healthcare). The dose rate at 500 mm distance from the source was 3.2 Gy/min, and 1.4 Gy/min at 750 mm distance.

### RNA interference

To deplete relevant target proteins, knockdown experiments were carried out using the following specific siRNAs: Negative control (siNC) (UUCUCCGAACGUGUCACGU), SKP2 (GUGAUAGUGUCAUGCUAAA), CDH1 (GGAUUAACGAGAAUGAGAA), CDC14B (GAUGCUACAUGGUUUAUA), CTIP (GCUAAAACAGGAACGAAUC), SKP1 (CGCAAGACCUUCAAUAUCA), CUL1 (GUUCAUAGCAGCCAGCCUG), USP4 (UUAAACAGGUGGUGAGAAA). P27 (GGAGCAAUGCGCAGGAAUAUU). (See Supplementary Methods for additional details). The siRNAs were delivered by nucleofection using the Nucleofector-2B device (Lonza). The program X-020 was used for A549 and RPE-1 cells; T-030 for 82-6-hTert, HFF hTert and AT hTert; X-001 for U2OS and M059K; Q-001 for HEK293. The knockdown efficiency was assessed by quantitating protein levels by western blot 48 h after nucleofection.

### Indirect immunofluorescence and image analysis

For immunofluorescence (IF) analysis, cells were grown on poly-l-lysine (Biochrom) coated coverslips. S-phase cells were labeled with 10 μM of 5-ethynyl-2′-deoxyuridin (EdU) for 30 min before irradiation. Cells were permeabilized in PBS supplemented 0.25% Triton X-100 (ROTH) for 5 min on ice. For RAD51 detection the pre-permeabilization step was omitted. Subsequently, cells were washed three times with PBS, and fixed in PFA solution, (3% paraformaldehyde and 2% sucrose), for 15 min at RT. After washing three times with PBS, samples were blocked in PBG solution (0.2% skin fish gelatin, 0.5% BSA fraction V, in PBS) overnight at 4 °C. The primary antibody against RPA (RPA70B) or RAD51 were diluted (1:300) in PBG solution. The cover slips were incubated at RT for 2 h and washed three times with PBS-T (0.05% Tween-20 in PBS). An Alexa Fluor-conjugated secondary antibody, anti-mouse IgG Alexa Fluor 488 (Thermo Fisher Scientific, A11001), was applied at 1:400 dilution for 1 h at RT. When necessary, the EdU signal was developed using an EdU staining kit (Thermo Fisher Scientific) according to the manufacturer’s instructions. Finally, cells were counterstained with 100 ng/ml DAPI (Thermo Fisher Scientific) at RT for 5 min and coverslips were mounted in PromoFluor antifade reagent (PromoCell). Scanning was carried out on a Leica TCS-SP5 confocal microscope (Leica Microsystems). G1 and G2 cells are EdU negative and can be discriminated from each other by comparing DAPI intensity (Fig. S1B). For each slide, 15 fields were scanned (~1000 nuclei) and the Z-stacks were processed using Imaris image analysis software (Bitplane). In total ~1600 cells were analyzed to obtain at least 100 EdU-negative, G_2_-phase cells for the quantification of parameters of interest. Data show means and standard deviations from two experiments

### Flow cytometry analysis of DNA end resection

For DNA end resection analysis using RPA70 detection, exponentially growing cells were pulse-labeled for 30 min with 10 µM EdU. After EdU incubation the growth medium was removed and cells were rinsed once with pre-warmed PBS, returned to growth medium and exposed to X-rays. At different times thereafter, cells were collected by trypsinization and unbound RPA was extracted by incubating the cell pellets for 2 min in ice-cold PBS containing 0.2% Triton X-100. Cells were spun-down for 5 min and pellets were fixed for 15 min with 3% PFA plus 2% sucrose dissolved in PBS. Cells were blocked with PBG blocking buffer overnight at 4 °C and incubated for 1.5 h with a monoclonal antibody raised against RPA70 (see above). Cells were washed twice with PBS and incubated for 1.5 h with a secondary antibody conjugated with AlexaFluor 488. Subsequently, EdU signal was developed using an EdU staining kit according to the manufacturer’s instructions. Finally, cells were stained with 40 µg/ml propidium iodide (PI, Sigma-Aldrich) at RT for 15 min. Three-parameter analysis was carried out with a flow cytometer (Gallios, Beckman Coulter). Similar to IF, EdU-negative G1 and G2 cells are discriminated by their PI intensity. For quantification, the Kaluza 1.3 software was used (Beckman Coulter). The experiments are replicated 3 times independently and a representative one is shown.

### Polyacrylamide gel electrophoresis (SDS-PAGE) and Western blotting

Cells were collected and washed twice in ice-cold PBS. Approximately 5 × 10^6^ cells were lysed for 30 min in 0.2 ml of ice-cold RIPA buffer (Thermo-Fisher) supplemented with Halt^TM^ phosphatase and protease inhibitor cocktails, as recommended by the manufacturer. Lysates were spun-down for 15 min at 12,000 × *g*, 4 °C and protein concentration was determined in the supernatants using the Bradford assay. Standard protocols for SDS-PAGE and immunoblotting were employed. Unless otherwise indicated, 50 μg RIPA whole-cell extract was loaded in each lane. Transfer of proteins onto nitrocellulose membranes and incubation with primary/secondary antibodies were performed according to standard procedures. Immunoblots were visualized by scanning membranes in an infrared scanner (Odyssey, Li-COR Biosciences). Western blots were processed using the brightness and contrast functions of the Odyssey software. The experiments are replicated 3 times independently and a representative one is shown.

### Pulsed-field gel electrophoresis

To analyze kinetics of DSB repair, Pulsed-field gel electrophoresis (PFGE) was performed. In this technique, the number of DSB present in cells is indirectly measured by the fraction of DNA released (FDR) out of the well into the lane of an agarose gel. Cells were trypsinized and suspended in serum free, HEPES-buffered medium (20 mM Hepes, 5 mM NaHCO_3_) at a concentration of 6 × 10^6^ cells/ml. Cells were then mixed with an equal volume of pre-warmed (50 °C) 1% low-melting agarose (Bio-Rad, Munich, Germany), and the cell suspension was pipetted into 3 mm diameter glass tubes. Agarose was allowed to solidify in ice, it was extruded from the glass tube and cut into 5-mm long blocks, which were irradiated in a Petri dish.

Cells in agarose plugs were lysed using the standard, high temperature lysis (HTL) protocol. Agarose blocks were pretreated in lysis buffer (10 mM Tris-HCl, pH7.6, 50 mM NaCl, 100 mM EDTA, 2% N-lauryl (NLS) and 0.2 mg/ml protease, added just before use) at 4 °C for 1 h, before lysis at 50 °C for 18 h. Subsequently, blocks with lysed cells were washed with washing buffer (10 mM Tris-HCl, pH 7.6, 50 mM NaCl, 100 mM EDTA) at 37 °C for 2 h, and digested with RNA digesting buffer (10 mM Tris-HCl, pH 7.6, 50 mM NaCl, 100 mM EDTA, and 0.1 mg/ml RNase, added just before use) at 37 °C for 2 h.

PFGE was carried out in gels cast with 0.5% molecular biology grade agarose (Bio-Rad), which was run in 0.5x TBE at 8 °C for 40 h. For separation of large molecular weight DNA, the electric field was pulsed and set at 50 V (1.25 V/cm) for 900 s in the forward direction and 200 V (5.00 V/cm) for 75 s in the reverse direction. After running, the gel was stained for 4 h with 1.6 µg/ml ethidium bromide and imaged using a fluor-imager (Typhoon 9400, Molecular Dynamics, Germany). FDR was analyzed using ImageQuant 5.2 (GE healthcare, Freiburg, Germany). All data shown represent the mean and standard deviation calculated from at least 6 determinations in 2 experiments.

### Enrichment of cells in G2 phase

We synchronized the 82-6-hTert and A549 cells at the G_1_/S transition using a single thymidine treatment. Cells were incubated in the presence of thymidine (2 mM) for 18 h. After washing using pre-warmed PBS twice, fresh growth medium was supplied to allow cells to progress in the cell cycle. At 7 h after release, G_2_-enriched cells were irradiated and collected for western blotting, either immediately or at the indicated times thereafter.

### Analysis of GC using GFP reporter cell lines

The U2OS-DR-GFP cell line (a gift from Dr. J. Stark) was specifically developed to report repair by GC of an I-*Sce*I induced DSB and was used as previously reported^42^. For the present of set of experiments, a similar reporter system was developed on the A549 cell line background and used according to the same protocols. For experiments, 2 × 10^6^ cells were transfected by nucleofection (Lonza) with 2 μg of the I-SceI expressing plasmid, pCMV3xNLS-I-SceI. At 24 h post transfection, cells were collected by trypsinization and GFP expression analyzed by flow cytometry (Gallios, Beckman Coulter) using a 488 nm argon laser. GFP emission was collected at FL1 using a 510BP filter. The frequency of repair events was calculated as the frequency of GFP-positive cells. Transfection efficiency was determined in each experiment using replicate cultures and 1 μg per 1 × 10^6^ cells of the pEGFP-N1 construct expressing GFP. Only experiments with transfection efficiency above 80% were analyzed further. When applicable, relevant knockdown of a protein was carried out 24 h before transfection of the I-SceI expressing plasmid. Data show means and standard deviations from three experiments.

### Cell survival determination

Cell survival was measured using the colony forming assay. Briefly, appropriate numbers of 82-6-hTert cells, as required to achieve about 100 colonies, were seeded in replicate 100 mm cell culture dishes and incubated for 10–14 d for colonies to develop. Subsequently, colonies were stained with crystal violet and counted. Data show means and standard deviations from three experiments

## Results

### SKP2 is required for resection in G_2_-phase

**Fig. 1:**
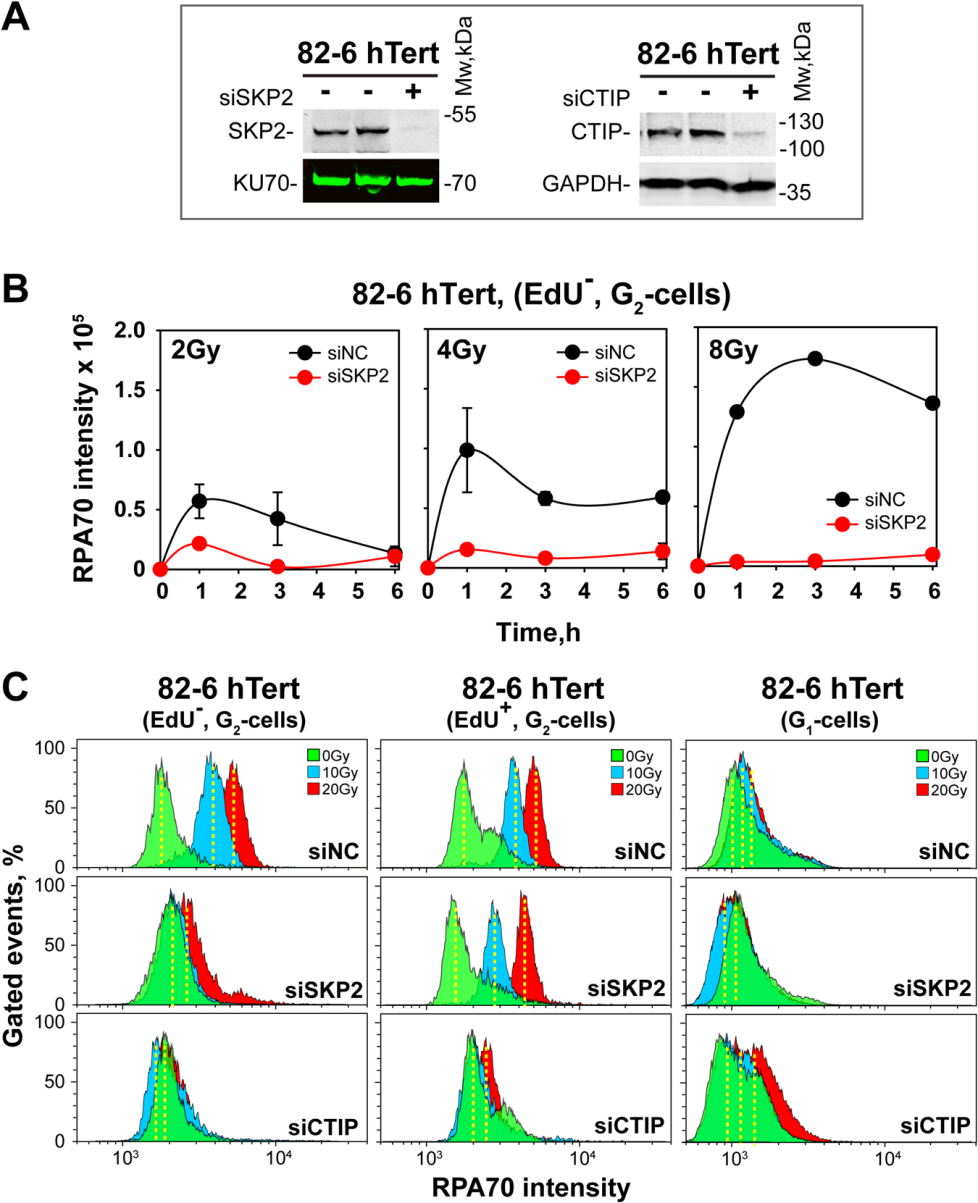
SKP2 is a positive regulator of DNA end resection in G_*2*_*-phase cells*. **a** Western blot (WB) analysis of SKP2 and CTIP in 82-6-hTert cells transfected with siRNA targeting these proteins. KU70 and GAPDH serve as loading controls. **b** Resection analysis in 82-6-hTert cells transfected with nonspecific control siRNA (siNC) or siRNA against SKP2 (siSKP2) after exposure to the indicated IR doses. Resection is measured by RPA70 intensity quantification using IF, specifically in EdU^−^, G_2_-cells. **c** Cell cycle specific quantification of resection by three-parametric flow cytometry in 82-6-hTert cells depleted for SKP2 or CTIP, exposed to 20 Gy and analyzed 3 h later. Results of cells irradiated in G_2_ (EdU^−^) or S (EdU^+^) and analyzed in G_2_ are shown (left panels), as well as results of cells irradiated in G_1_ and analyzed in G_1_ (right panels). See text for details.

We investigated contributions of SCF^SKP2^ to resection at DSBs induced by ionizing radiation (IR) by depleting SKP2. Our recent work shows profound differences in the regulation of resection between cells irradiated in S- versus G_2_-phase, as well as at low versus high IR-doses^43,44^. Therefore, we conducted cell-cycle- and IR-dose-dependent analysis. At low IR-doses, cell cycle-dependent analysis is possible by IF. S-phase cells are labelled with EdU^44^ and resection measured by quantitating chromatin-bound RPA in EdU-negative (EdU^−^) cells, in the G_2_-phase compartment (Fig. S1A). This compartment comprises cells irradiated in G_2_-phase that remain in G_2_ at the time of analysis. Fig. S1A shows the gates applied, while Fig. S1B representative G_2_-phase nuclei of 82-6-hTert cells, 3 h after exposure to 0–8 Gy. The marked increase in RPA signal after IR, reflects robust resection. Transfection of cells with siRNAs targeting SKP2 causes its depletion 48 h later, compared to cells transfected with a non-targeting, control siRNA (siNC) (Fig. 1a, Fig. S1B). Strikingly, SKP2 depletion suppresses resection at all IR doses investigated (Fig. 1b, Fig. S1B).

To extend resection-analysis to higher IR-doses and additional cell cycle phases, we introduced a flow-cytometry-based (FC) method^43,44^ and similar analysis approaches (Fig. S1C). The EdU^-^ compartment in the G_2_–phase-gate comprises cells irradiated and remaining in G_2_-phase. The EdU^+^ compartment in the S-phase-gate comprises cells irradiated in S-phase that remain in S-phase, while the EdU^+^ compartment in the G_2_–phase-gate ((EdU^+^-G2), cells irradiated in S-phase that progress to G_2_–phase. The G_1_-phase compartment with EdU^-^ cells reflects cells in G_1_ at irradiation that remain in G_1_.

Figure S1C shows representative FC-data and the gates applied, while Fig. S1D illustrates RPA signal distribution in G_2_-phase, 3 h after exposure of 82-6-hTert cells to 0 or 10 Gy, for EdU^+^ and EdU^−^ cells. Increase in RPA signal after IR documents robust resection in both cell populations, quantification of which is readily possible up to 20 Gy (Fig. 1c, siNC-top-panels). Included in Fig. 1c are also results of G_1_-cells. Since resection is low in G_1_-phase, the RPA signal is lower even in non-irradiated cells; this low signal changes only marginally after exposure to IR, compromising analysis of the SKP2-effect on resection. Resection analysis during S-phase is also compromised by DNA replication that generates high RPA background signal (results not shown, but see Fig. S1C). Therefore, we focus here on G_2_-phase cells, but include cells irradiated in G_2_–phase^44^, as well as cells irradiated in S-phase that enter G_2_-phase during the postirradiation incubation period^43^.

SKP2 depletion causes nearly complete suppression of resection at all IR-doses in EdU^−^ cells (Fig. 1c, left panels) in agreement with Fig. 1b. Strikingly, in EdU^+^ cells, SKP2 depletion only marginally reduces resection (Fig. 1c, middle panels). The suppressive effect of SKP2-depletion on resection in G_2_-irradiated cells persists for at least 6 h (Fig. S2A, B). Predictably, CTIP-depletion practically eliminates resection, both in EdU^-^ as well as in EdU^+^ G_2_-phase cells (Fig. 1c, lower panels), underscoring the cell cycle specificity of the SKP2-effect. The reduction in resection by CTIP depletion is not a consequence of cell cycle redistribution (Fig. S2C). We conclude that in 82-6-hTert cells, resection in G_2_-phase requires SKP2 only when cells are irradiated in G_2_-phase, and that this effect holds for high and low doses of IR. The following experiments analyze therefore effects at a single IR-dose. Notably, Fig. S2D–F show that the effect of SKP2 on resection is independent of P27. We surmise that the suppression of resection observed after SKP2 knockdown is predominantly mediated by the depletion of CTIP, and that activity-reduction of residual CTIP via P27-mediated suppression of CDK1 activity is not detectably contributing.

The dependence of resection on SKP2 in G_2_-phase cells is not a peculiarity of 82-6-hTert cells. It is observed at similar levels in HFF-hTert human fibroblasts (Fig. S3A), as well as in human lung carcinoma A549 cells (Fig. S3B). Complete dependence on SKP2 also show human glioma M059K cells (Fig. S3C), while human embryonic kidney (HEK) 293 cells show a marked but incomplete inhibition of resection upon SKP2 knockdown (Fig. S3D). On the other hand, retinal pigmented epithelial RPE1-hTert cells show suppression of resection after SKP2 knockdown only at early times after IR (Fig. S4A). Strikingly, the widely used human bone osteosarcoma U2OS cells show no detectable effect on resection upon SKP2 depletion (Fig. S4B). Finally, AT-hTert cells show complete suppression of resection after SKP2 knockdown, despite the altered resection kinetics associated with the AT defect (Fig. S4C)^44^. In all cell lines, SKP2 depletion fails to suppress G_2_-phase resection, when cells are irradiated in S-phase (results not shown). Collectively, we conclude that SCF^SKP2^ is a general, albeit not universal, positive regulator of resection in G_2_-phase, without evident IR-dose-dependence, or dependence on ATM^45^.

### SKP2 maintains CTIP levels in G_2_-phase

We inquired whether the suppression of resection after SKP2 depletion is mediated by regulatory adaptations of the levels of resection proteins. Because the effect is limited to G_2_-phase cells, we employed treatment with thymidine to generate G_2_-phase-enriched populations of 82-6-hTert cells (Fig. S5A). Figure S5B, C show representative cell cycle distributions at different stages in the synchronization procedure, as well as after SKP2 depletion and irradiation. Populations showing satisfactory enrichment in G_2_-phase, with acceptable reproducibility, are obtained 6–8 h after thymidine-block-release.

**Fig. 2:**
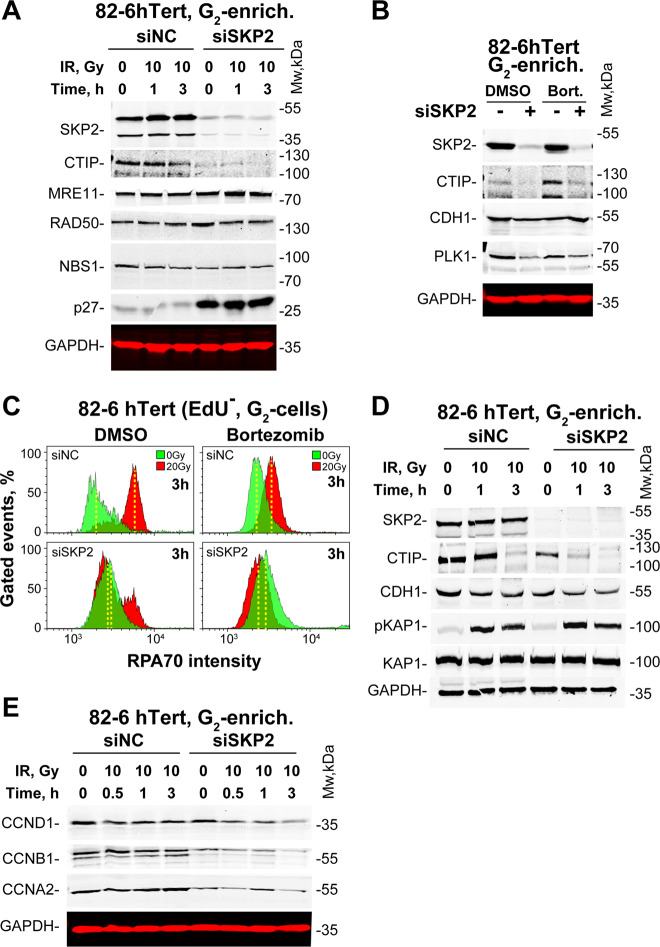
SKP2 regulates resection by stabilizing CTIP. **a** WB analysis of CTIP, MRE11, RAD50, and NBS1 in SKP2 depleted and control, G_2_-enriched 82-6-htert cells. GAPDH serves as a loading control. **b** WB analysis of CDH1 and PLK1 in SKP2 depleted and control G_2_-enriched 82-6-hTert cells after treatment with the proteasome inhibitor bortezomib, 2 µM, 2 h. **c** Resection analysis in G_2_, as described in 1C, for 82-6-hTert cells irradiated (20 Gy) in G_2_ and treated or not with bortezomib. **d** WB analysis of CDH1 and pKAP1 in G_2_-enriched 82-6-hTert cells after SKP2 knock-down and exposure to 0 or 10 Gy. **e** WB analysis of Cyclin D1 (CCND1), Cyclin B1 (CCNB1) and Cyclin A (CCNA2) in G_2_-enriched and SKP2-depleted 82-6-hTert or control cells after exposure to 0 or 10 Gy.

Fig. 2a shows resection-related proteins in G_2_-phase cells exposed to 0 or 10 Gy and analyzed 1 or 3 h later. Results of cells transfected with siNC or siSKP2 48 h before IR are shown. It is evident that siSKP2 efficiently depletes SKP2 in G_2_-enriched populations as well. Notably, SKP2 depletion also causes a marked depletion of CTIP, while MRE11, RAD50 and NBS1 remain unchanged. Predictably P27, a target of SCF^SKP2^, is stabilized after SKP2 knockdown, providing functional proof of SCF^SKP2^ activity-inhibition. We conclude that SCF^SKP2^ regulates resection by stabilizing CTIP.

CTIP is regulated by APC/C^CDH1^-mediated ubiquitination^22,41,46^. We examined therefore the effects of the proteasome inhibitor bortezomib^47–50^ on CTIP levels. Treatment with bortezomib (2 µM, 2 h) increases CTIP levels in G_2_-phase cells (Fig. 2b), but causes unexpectedly a decrease rather than increase in resection after IR (Fig. 2c, upper panels), suggesting complex mechanistic inputs to this endpoint. Strikingly, even in bortezomib-treated cells, SKP2-knockdown depletes elevated CTIP in non-irradiated cells (Fig. 2b) and eliminates residual resection after IR (Fig. 2c). Notably, in this experimental setting, known proteasome targets, such as CDH1 and PLK1, remain largely unaffected, emphasizing regulatory complexity in their maintenance (Fig. 2b). We conclude that stabilization of CTIP by SCF^SKP2^ is regulated by processes prior to its proteasomal degradation.

The well-established, intimate crosstalk between SCF^SKP2^ and APC/C^CDH1^ and the documented degradation of CTIP by APC/C^CDH1^ prompted us to inquire whether SCF^SKP2^ functions in coordination with APC/C^CDH1^ to regulate resection after IR. Normally, SCF^SKP2^ contributes to APC/C^CDH1^ activation late in G_2_-phase; too late to contribute to DDR activation and resection in G_2_-phase, without additional regulatory inputs. Notably, APC/C^CDH1^ is prematurely activated after IR and helps to enforce the G_2_-checkpoint by the translocation from nucleolus into the nucleoplasm of CDC14B, to mediate PLK1 degradation and WEE1 and Claspin stabilization^38,51,52^. We postulated therefore that in an environment where APC/C^CDH1^ is activated, SCF^SKP2^ will function to protect CTIP from its otherwise inevitable degradation.

Fig. 2d shows that indeed, SKP2 depletion reduces CtIP, even without IR. When SKP2 is present, CTIP levels remain high up to 1 h and decrease only at 3 h, providing time for CTIP to sustain resection. Notably, SKP2 depletion causes marked CTIP degradation at 1 h after IR and CTIP is undetectable at 3 h, in line with the observed inhibition of resection. We conclude that SCF^SKP2^ protects CTIP from APC/C^CDH1^-mediated degradation and that this protection is enhanced after IR. The robust residual CDH1 levels detected under all conditions examined in this experiment (Fig. 2d) underscore the degradation potential of APC/C^CDH1^ towards CTIP. The activation of APC/C^CDH1^ in G_2_-phase cells after SKP2 knockdown is also functionally demonstrated by the reduction in the levels of known APC/C^CDH1^ substrates, such as cyclin B1 (CCNB1), and cyclin A (CCNA2), and the potentiation of this effect after IR (Fig. 2e). Notably, levels of cyclin D1 (CCND1), a protein not targeted by APC/C^CDH1^, are less affected.

**Fig. 3:**
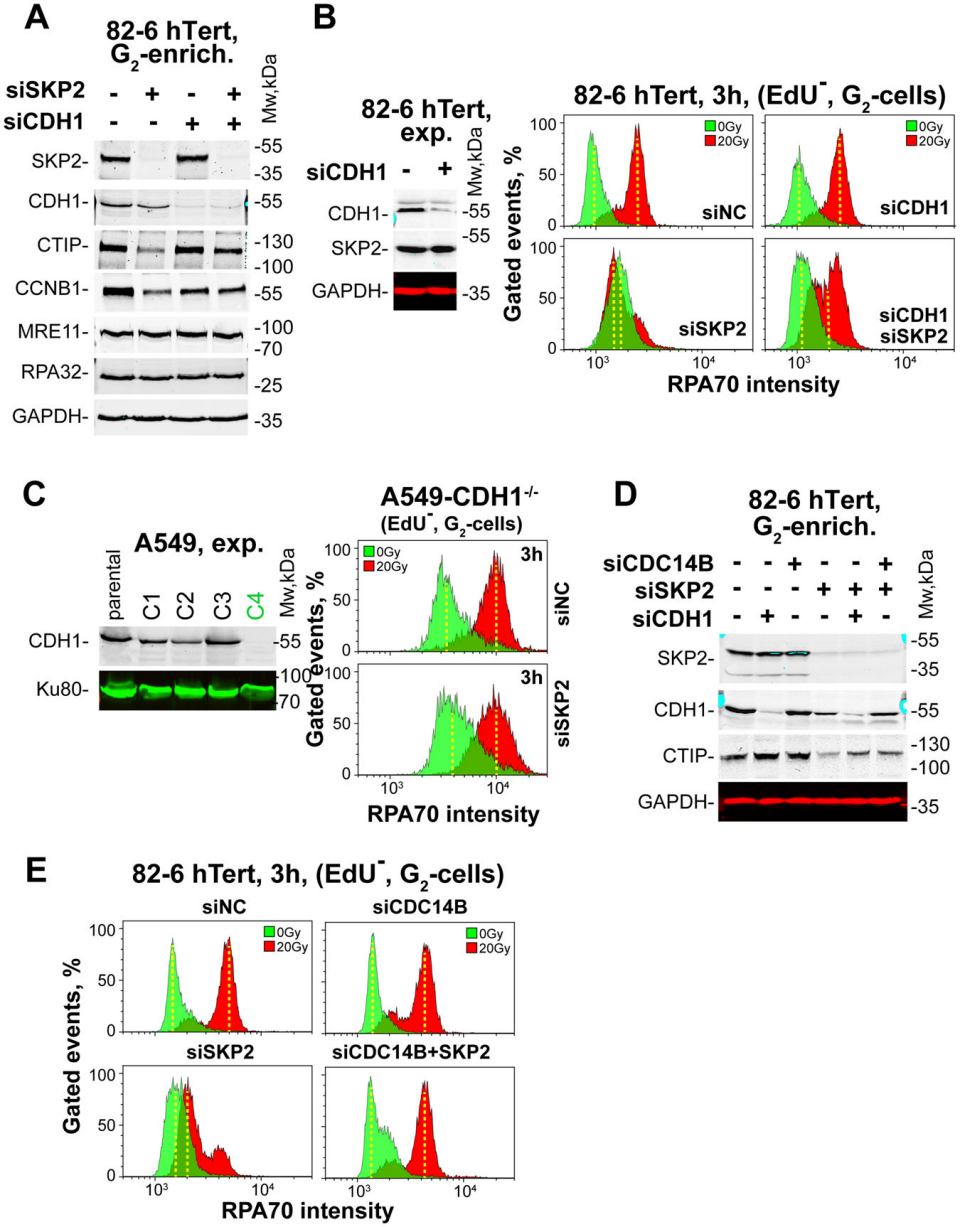
CDH1 depletion restores CTIP degradation and suppression of resection induced by SKP2 depletion. **a** WB analysis of SKP2, CDH1, CTIP, CCNB1, MRE11, and RPA32 in G_2_-enriched 82-6-hTert cells after depletion of SKP2 or CDH1, alone or in combination. GAPDH serves as a loading control. **b** Resection analysis in EdU^−^ G_2_ 82-6-hTert cells, as described in Fig. 1c, after exposure to IR (20 Gy) after depletion of SKP2 or CDH1, alone or in combination. The dual peaks in these experiments are suggestive of two subpopulations one of which is effectively transfected and shows complete response and a second one less efficiently transfected that shows no response. **c** WB validation of CDH1 knockout in the C4 clone of A549 cells. Resection analysis in G_2_, as described in 1C, for A549/C4 cells irradiated (20 Gy) in G_2_ after depletion of SKP2. **d** WB analysis of SKP2, CDH1 and CTIP after knockdown of CDC14B, SKP2, or CDH1 alone or in combination in G_2_-enriched 82-6-hTert cells. **e** Resection analysis in EdU^−^ G_2_ 82-6-hTert cells exposed to IR (20 Gy) after knockdown of CDC14B or SKP2, alone or in combination.

To further test the model of dynamic interaction between SCF^SKP2^ and APC/C^CDH1^ in regulating CTIP and resection in G_2_-phase, we examined parallel depletion of SKP2 and CDH1. Figure 3a shows that while SKP2-knockdown depletes CTIP in non-irradiated cells, dual depletion of SKP2 and CDH1 rescues CTIP. CCNB1 responds similarly, while MRE11 and RPA32 remain unaffected. Also resection is partly restored after combined depletion (Fig. 3b). As with CDH1 depletion, CDH1 inhibition using Tosyl-l-Arginine Methyl Ester (proTAME), is ineffective on resection^41^ (Fig. S6A).

Similar results are obtained in A549 cells and are summarized in Figs. S3B and S6B–D. We also employed CRISPR/Cas9 technology to generate a CDH1 knockout mutant in A549 cells. Figures 3c and S6E, F confirm the knockout by the absence of the protein in the selected clone (C4). Notably, SKP2 knockdown leaves resection unaffected in this mutant confirming that the SKP2-dependent degradation of CTIP and the associated suppression of resection in G_2_-phase require the activity of APC/C^CDH1^.

Since activation of APC/C^CDH1^ in G_2_-phase after IR requires the release of CDC14B from the nucleolus, we tested the effect of its knockdown. CDC14B knockdown has no effect on resection (Fig. 3d, e), as also observed for CDH1 knockdown. However, combined CDC14B and SKP2 knockdown reverses the effect on resection and CTIP levels of SKP2 knockdown (Fig. 3e, d), demonstrating that APC/C^CDH1^ activity requires CDC14B.

**Fig. 4:**
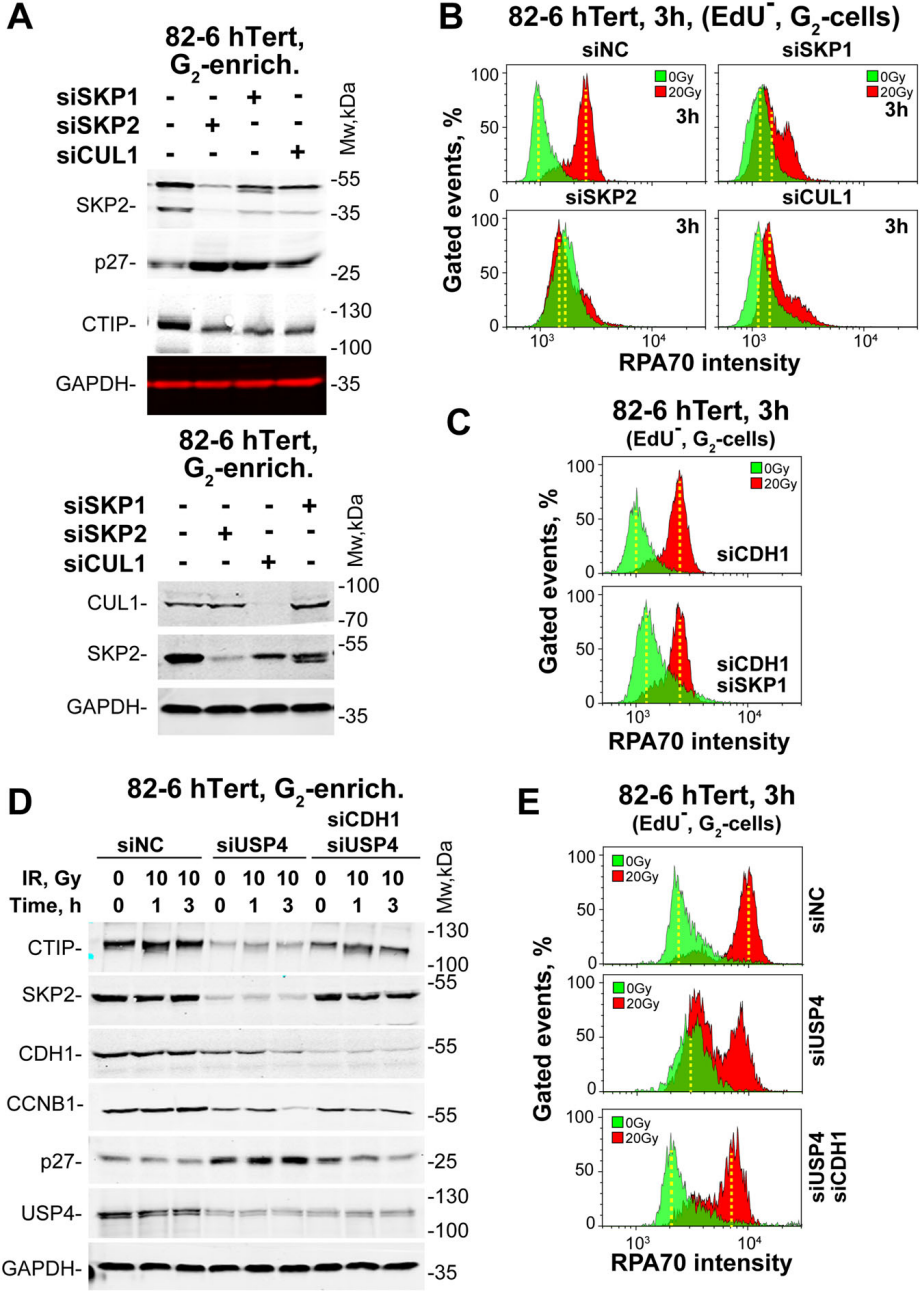
Effects of knockdown of SCF^SKP2^ complex components on resection. **a** WB of CTIP in G_2_ enriched 82-6-hTert cells after depletion of SKP1, SKP2 or CUL1. P27 and SKP2 levels trace the efficiency of the SKP2 knockdown. GAPDH serves as a loading control. **b** Resection analysis, as described in Fig. 1c, for 82-6-hTert cells irradiated (20 Gy) in G_2_ after depletion of SKP1, SKP2 or CUL1. **c** As in Fig. 4b for cells depleted of CDH1, or CDH1 + SKP1. **d** WB of CTIP, SKP2, CDH1, CCNB1, P27, and USP4 in G_2_ enriched 82-6-hTert cells depleted of USP4 and/or CDH1, exposed to 0 or 10 Gy and analyzed 1 or 3 h later. **e** Resection analysis at 3 h in G_2_ 82-6-hTert cells irradiated (20 Gy) in G_2_ after depletion of USP4 or USP4 + CDH1.

We inquired how SKP2 affects APC/C^CDH1^ and whether its effect requires all components of the SCF^SKP2^ complex. Figure 4a shows that knockdown of two essential components of SCF^SKP2^, SKP1 and CUL1, deplete CTIP similar to SKP2 knockdown. Notably, knockdown of either protein markedly reduces resection as well (Fig. 4b). Here again, knockdown of SKP1 and CDH1 rescues resection (Fig. 4c).

DNA damage induced activation of APC/C^CDH1^ in G_2_-phase induces the degradation of its natural G_1_-phase substrates. Since some of these substrates are DDR components, their degradation is counteracted by deubiquitylation. Thus, USP28 deubiquitinates Claspin to enable the activation of a CHK1-dependent G_2_-checkpoint^38^, and protects checkpoint-mediators to facilitate apoptosis^53^. Also USP4 is implicated in G_2_-phase-related events and shown to promote resection and GC through interactions with CTIP and MRN^54,55^. Finally, other reports implicate USP8 in DDR^56^.

We inquired whether CTIP is protected by deubiquitinases from degradation after IR. Figure 4d, e summarize results with USP4, as USP8 and USP28 failed to generate consistent results in all endpoints. USP4 knockdown, although incomplete, reduces CTIP levels (Fig. 4d), suggesting that it normally protects CTIP from degradation. Notably, under these conditions, resection is abolished in 60% of cells (Fig. 4e), providing functional proof for this protection. Surprisingly, USP4 knockdown also reduces the levels of SKP2 and CDH1 pointing to distortions in the dynamic equilibrium between APC/C^CDH1^ and SCF^SKP2^. However, while increase in P27 documents suppression of SCF^SKP2^ activity, the reduction in CCNB1 levels demonstrates residual APC/C^CDH1^ activity to mediate CTIP depletion after SCF^SKP2^ inhibition. Notably, combined depletion of USP4 and CDH1 reverts the effects on these proteins (Fig. 4d) and restores resection (Fig. 4e). We conclude that USP4 protects CTIP from degradation and helps to maintain resection. However, it remains to be elucidated whether this is a direct effect on CTIP deubiquitination, or an indirect effect mediated by SCF^SKP2^ inactivation. Indeed, it has been reported that USP4 regulates resection by an unknown mechanism^55^. Collectively, we conclude that in cells irradiated in G_2_-phase, SCF^SKP2^ suppresses the activity of APC/C^CDH1^ specifically towards CTIP.

### SKP2 knockdown suppresses DSB-repair in G2-phase cells

**Fig. 5:**
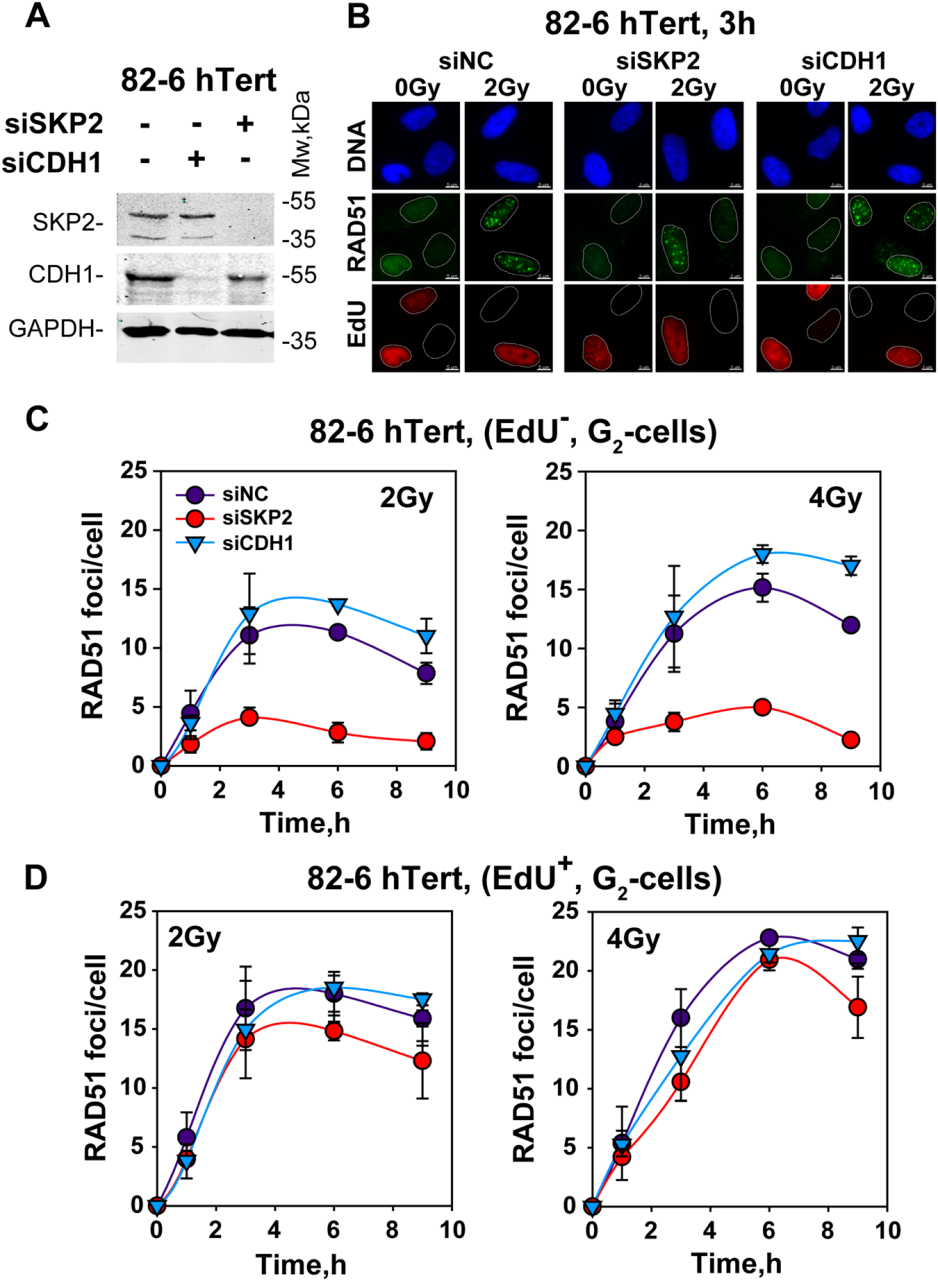
SKP2 depletion suppresses GC, specifically in cells irradiated in G_2_-phase. **a** WB showing depletion of SKP2 or CDH1 in 82-6-hTert cells. **b** Representative IF images showing RAD51 foci, as well as EdU and DAPI signals, in 82-6-hTert cells depleted of SKP2 or CDH1, exposed to 0 or 2 Gy or IR and analyzed 3 h later. **c** Kinetics of RAD51 foci formation, in 82-6-hTert cells depleted of SKP2 or CDH1 exposed to 2 or 4 Gy or IR in G_2_-phase and analyzed also in G_2_. **d** As in **c** for cells irradiated during the S-phase but analyzed in G_2_ (see text for details).

To examine the effect of SKP2 depletion on GC, we measured RAD51 foci formation in EdU^−^ and EdU^+^, G_2_-phase cells. Figure 5b shows representative images and Fig. S7 details of the cell cycle-specific analysis employed. Figure 5c shows that in EdU^−^ cells exposed to 2 or 4 Gy, RAD51 foci form robustly confirming active GC. While depletion of CDH1 (Fig. 5a) slightly increases this response, depletion of SKP2 causes a pronounced suppression demonstrating inhibition of GC. Strikingly, this inhibition is specific for cells irradiated in G_2_-phase, as it is much reduced in cells irradiated in S-phase (Fig. 5d).

We also used cell lines harboring the DR-GFP reporter to examine in a functional manner the role of SKP2 in GC. In a cell line we generated using A549 cells, expression of I-*Sce*I causes a marked increase in GFP-positive cells that is strongly inhibited after SKP2 knockdown (Fig. S8A). Strikingly, but predictably from the lack of effect on resection (Fig. S4B), SKP2 knockdown in U2OS-DR-GFP cells has only a marginal effect on GC (Fig. S8B).

**Fig. 6:**
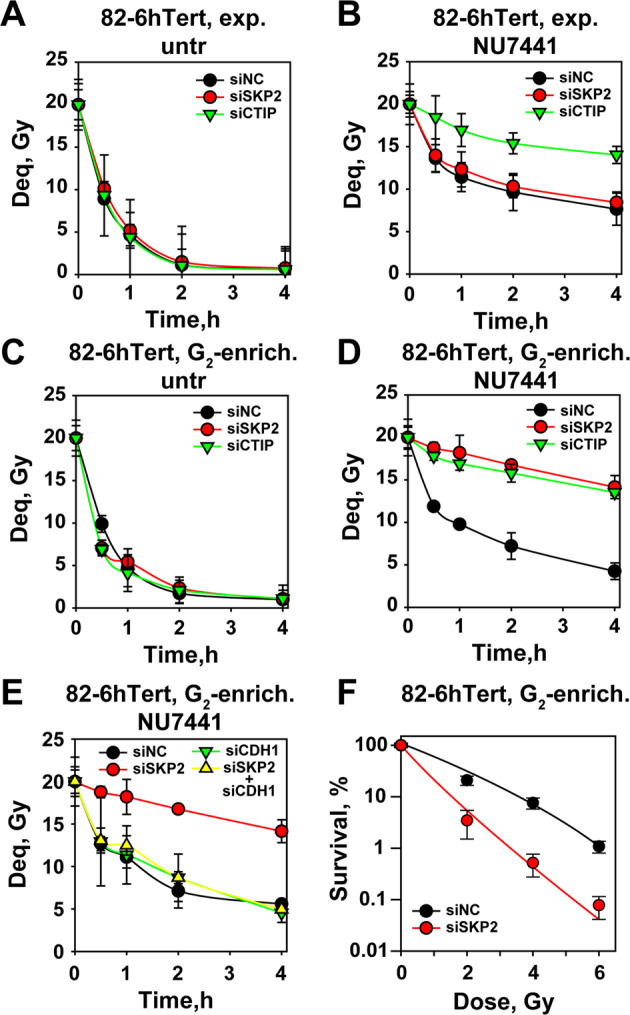
SKP2 depletion suppresses alt-EJ in G_*2*_-phase irradiated cells. **a** PFGE analysis of DSB repair in SKP2 or CTIP depleted exponentially growing 82-6-hTert cells after exposure to 20 Gy. **b** As in **a** for cells treated with the DNA-PKcs inhibitor NU7441, to reveal alt-EJ. **c** As in **a** for G_2_ enriched 82-6-hTert cells. **d** Same in **b**, but for G_2_-enriched 82-6-hTert cells. **e** As in **d** but including combinations with cells depleted of CDH1, as well as CDH1 + SKP2. **f** Survival of G_2_-enriched 82-6-hTert cells 48 h after treatment with siNC or siSKP2.

To also investigate the effect of SKP2 on c-NHEJ and alt-EJ, we employed PFGE to analyze DSB repair. Figure 6a shows that SKP2 or CTIP knockdown have no effect on DSB processing in actively growing 82-6-hTert cells, when tested under conditions mainly allowing analysis of c-NHEJ. When actively growing 82-6-hTert cells are treated with the DNA-PKcs inhibitor NU7441, c-NHEJ is suppressed and residual DSB processing reflects the function of resection-dependent pathways including alt-EJ and SSA. GC is suppressed at the high IR-doses employed here^57^. Suppression of resection by depletion of CTIP in NU7441 treated cells, strongly inhibits DSB processing confirming its function on resection-dependent DSB repair pathways (Fig. 6b). Notably, depletion of SKP2 is without effect (Fig. 6b). Retrospectively, this is a predictable outcome because SKP2 only functions to suppress resection in G_2_-phase cells, which only represent a small percentage in the actively growing populations used in this experiment.

We conducted therefore experiments with G_2_-phase-enriched cells. Figure 6c shows that depletion of CTIP or SKP2 is ineffective even in G_2_-cells when c-NHEJ is functional. Notably, when G_2_-phase-enriched cells (Fig. S8C) are treated with NU7441, depletion of SKP2 causes an effect practically indistinguishable from that of CTIP knockdown, confirming the above results and demonstrating the function of resection-dependent, DSB-repair pathways under these conditions (Fig. 6d).

When CDH1 is depleted, the activity of resection-dependent pathways remains unchanged although SKP2 knockdown suppresses, as expected, DSB processing (Fig. 6e). Strikingly, combined depletion of SKP2 and CDH1 fully rescues this inhibition, in line with the restoration of resection seen above. Finally, SKP2 knockdown markedly radiosensitizes G_2_-phase cells to IR (Fig. 6f), emphasizing the physiological significance of the described effects. We postulate that the strong radiosensitization observed in G_2_-phase after SKP2 knockdown reflects inhibition of GC, rather than inhibition of alt-EJ. We conclude that SKP2 sustains resection in irradiated cells and promotes resection-dependent DSB-processing and cell survival, but only in the G_2_-phase of the cell cycle.

## Discussion

### Dynamic regulation of resection by the SCF^SKP2^-APC/C^CDH1^ module

Our results demonstrate that SCF^SKP2^ is a strong positive regulator of resection in G_2_-phase, acting by protecting CtIP from APC/C^CDH1^ mediated degradation. CTIP is constitutively degraded by APC/C^CDH1^ in G_1_–phase to generate the low-resection-activity environment present in this phase, and in irradiated G_2_-phase cells to suppress hyper-resection at DSBs^41^. The integration of APC/C^CDH1^ to the SCF^SKP2^-dependent regulation of CTIP, defines a dynamic module delicately regulating resection after IR. Thus, the complete set of cell cycle regulators, including CDKs (and cognate-CKIs) and SCF^SKP2^-APC/C ubiquitin ligases, feature also as central regulators of resection and thus of DSB-repair pathway-choice.

Interestingly, however, the temporal coordination between SCF^SKP2^ and APC/C^CDH1^ is different in cell cycle^36,39^ and resection-regulation. In the unperturbed cell cycle, the SCF^SKP2^-APC/C protein degradation module enforces directionality through the degradation of selected substrates, at well-defined cell cycle transitions. Thus, active SCF^SKP2^ suppresses the activity of APC/C, while active APC/C suppresses the activity of SCF^SKP2^. This includes the suppression of SCF^SKP2^activity in G_1_ by APC/C-mediated SKP2-degradation, as well as the suppression of APC/C activity by Cdk-mediated phosphorylation of CDH1 from late-G_1_ to late G_2_-phase following SCF^SKP2^ activation^35^. SKP2 enables entry into S-phase by also targeting cell cycle inhibitors, such as P27 and P21^36,58^. APC/C^CDH1^ is re-activated after mitosis and remains active in G_1_, where it degrades substrates required for DNA replication. All these aspects of mutual regulation show a clear temporal separation, often by many hours. In contrast, SCF^SKP2^ and APC/C^CDH1^ regulate resection at DSBs simultaneously, acting on the same target: CTIP.

As observed here and as reported previously, APC/C^CDH1^ is activated in irradiated G_2_-cells by CDC14B-mediated dephosphorylation of CDH1. Such activation would naturally cause degradation of its targets, including CtIP, precisely in a phase of the cell cycle, where all resection-dependent DSB-repair pathways are programmed for maximum activity. The SCF^SKP2^-dependent stabilization of CtIP reported here for the first time, prevents this adverse consequence and rescues resection-dependent DSB-processing. CtIP-rescue may involve a direct dampening of APC/C^CDH1^ activity, or a SCF^SKP2^-dependent activation of USP4, similar to Claspin stabilization by USP28^38,59^.

The positive regulation of resection by SCF^SKP2^ is essential for DSB-repair pathway-choice^3,4^. Indeed, GC and alt-EJ are suppressed after inhibition of SCF^SKP2^. Notably, this effect is confined to cells irradiated in G_2_-phase of the cell cycle. Moreover, it is restricted to cell lines showing SCF^SKP2^ dependence in the regulation of resection. Finally, and as expected, the suppression of resection-dependent DSB-processing in G_2_-phase causes a marked increase in the sensitivity of cells to IR-induced killing.

Additional connections with DDR have been reported for APC/C–SCF. Thus, APC/C is connected to DSB-induced checkpoint responses and degrades PLK1 to suppress progression to mitosis^38,59^ and to stabilize Claspin and WEE1 after IR, reducing thus genotoxic stress^38,60,61^. APC/C^CDH1^ is also required for G_1_ cell cycle arrest^62^. Interestingly, SCF^CyclinF^ negatively regulates resection by targeting EXO1 in response to replication stress^63^, and SCF^SKP2^ interacts with NBS1 to regulate DSB-processing, but the effect of this modification on resection has not been investigated^45^. CDC25A is degraded by SCF^β-TrCP^ to support G_2_-checkpoint activation^64^, and SCF^β-TrCP^ contributes to checkpoint recovery by degrading PLK1-phosphorylated Claspin^65,66^. Finally, SCF^β-TrCP^ regulates translation during G_2_-checkpoint recovery^67^.

### Regulation of resection by SCF^SKP2^-APC/C^CDH1^ is cell cycle dependent

Resection inhibition by SKP2 knockdown uncovers a direct involvement of SCF^SKP2^ in DSB repair pathway selection, albeit in a cell line dependent manner (Figs. S3 and S4). Moreover, the effect is strictly confined to cells irradiated in G_2_-phase. Cells irradiated in S-phase resect efficiently when they reach G_2_-phase, but this resection does not require SCF^SKP2^ activity. Such profound mechanistic shifts in the regulation of DDR between cells irradiated in S- and G_2_-phase follows step with our recent work analyzing the wiring between DNA-PKcs, ATM and ATR in the regulation of resection and G_2_-checkpoint activation^43,44^. Thus, in cells exposed to low IR-doses in G_2_-phase, resection and checkpoint are regulated epistatically by ATM and ATR, whereas at high IR-doses ATM and ATR can act independently^44^. Strikingly, when cells are irradiated during S-phase, the checkpoint activated in G_2_-phase is regulated exclusively by ATR, independently of IR-dose, and resection is independent of ATR activity^43^. In all cases DNA-PKcs integrates to the ATM/ATR module to suppress hyperresection.

The G_2_-phase-specificity in the regulation of resection by SCF^SKP2^ can be rationalized by the observation that resection in S/G_2_ relies on the transient (1–3 h) stabilization of CTIP against APC/C^CDH1^-mediated degradation, whose activity is constitutively low in S-phase obviating SCF^SKP2^-stabilization. Regardless, our previous observations and the results presented here strongly suggest that different mechanisms regulate pathway-choice and checkpoint during S and G_2_-phase and call for cell-cycle-specific analysis.

APC/C is a documented tumor suppressor, whereas SCF^SKP2^ an oncogene. Indeed, overexpression of SKP2 is found in a variety of human cancers promoting progression, invasion and metastasis^36,68^, while deficiency inhibits these processes^69,70^. SKP2 deficiency also causes polyploidy and micronuclei formation^71^, indicating that SKP2 is required for the maintenance of genomic stability. Our results explain how this might be possible. The functions SCF^SKP2^ on resection add to those of cell cycle regulation and accentuate its oncogenic properties by promoting genomic instability and suppressing DDR, which is considered a major tumor suppressor^72^.

## Supplementary information

Supplementary Information

Supplementary Information

Supplementary Information

Supplementary Information

Supplementary Information

Supplementary Information

Supplementary Information

Supplementary Information

Supplementary Information

Supplementary Information
