# Supplementary material for: SCFSKP2 regulates APC/CCDH1-mediated degradation of CTIP to adjust DNA-end resection in G2-phase
Source: Cell Death Dis. 2020 Jul 18;11(7):548. doi: 10.1038/s41419-020-02755-9 (PMC7368859; doi:10.1038/s41419-020-02755-9)
Supplement: Supplementary file 1 — Supplementary Information [file 41419_2020_2755_MOESM1_ESM.docx]

## Supplementary Figure Legends

**Figure S1**: (A) Representative dot plots showing events of EdU vs DAPI intensity in 82-6 hTert cells exposed to 2 Gy after treatment for 48 h with siNC or siSKP2 and analyzed 3 h later. The gates employed to define EdU negative populations of cells (EdU-) are indicated in green color. This gate is placed to select cells in G2-phase, defined by DAPI intensity. (B) Representative immunofluorescence images of EdU^-^, G2-phase, 82-6 hTert cells, transfected with siNC or siSKP2, exposed to different doses of IR and analyzed 3 h later. For analysis about 10 fields of view, containing at least 500cells are captured by confocal microscopy and images quantified by Imaris 9.3 software (Bitplane). Data are plotted using a graphics software (Orange). (C) Outline of the three-parametric flow cytometry analysis employed to quantitate resection in G2-irradiated cells exposed to high IR doses. Upper panels: Dot plots showing RPA70 *versus* PI signals. Lower panels: Dot plots showing EdU *versus* PI signals. Shown in these panels are also the gates applied for quantitating resection at different times after IR in cells at different phases of the cell cycle. (D) RPA70 signal in non-irradiated (0 Gy, green) or irradiated (10 Gy, red) G2-phase cells that are either EdU^+^ or EdU^-^.

**Figure S2**: (A) Resection kinetics measured by flow cytometry in G2-phase, 82-6 hTert cells irradiated (20 Gy) 48 h after transfection with siNC or siSKP2 and analyzed 3 h later. The gates shown on Figure S1C are applied for selecting the EdU^-^, G2-phase cells. (B) Quantification of RPA70 signal from three experiments as shown in (A) (P<0.05 in all there 3 points). The RPA70 signal is normalized to the RPA70 signal detected in non-irradiated cells. Data represents means and standard deviations.

(C) Representative cell cycle distributions of 82-6 hTert cells depleted of CTIP or SKP2 for 48 h, exposed to the indicated doses of IR and analyzed 3 h later. (D) Resection analysis in 82-6 hTert cells in G2, for 82-6 hTert cells irradiated (20 Gy) in G_2_ after depletion of SKP2 or P27, alone or in combination. (E) WB analysis of P27 and SKP2 in 82-6 hTert cells transfected with siRNA targeting these proteins. β-ACTIN serves as loading control. (F) Cell-cycle distribution after depletion of SKP2 or P27, alone or in combination, determined by PI staining.

**Figure S3**: (A) Resection kinetics of HFF hTert cells treated with siNC or siSKP2 for 48 h and exposed to 20 Gy. EdU- cells are specifically analyzed. Other details as in Figure S1. (B) As in

1. for A549 cells. (C) As in (A) for M059K cells. (D) As in (A) for HEK293 cell.

**Figure S4**: (A) As in Fig. S3A for RPE-1 cells (B) As in Fig. S3A for U2OS cells. (C) As in Fig. S3A for ATM deficient, AT hTert cells.

**Figure S5**: (A) Schematic representation of the synchronization procedure employed using treatment with 2 mM thymidine. Cells are allowed to grow for one division and subsequently thymidine is added for 18-20 h depending on the cell line. Cells are released from thymidine block by washing with pre-warmed PBS and are irradiated when they reach G2-phase. (B) Representative cell cycle distributions at different stages in the synchronization procedure. (C) As in Figure S5B, for cells depleted of SKP2.

**Figure S6**: (A) Resection analysis in 82-6 hTert cells treated with the CDH1 inhibitor proTAME, 20 µM, 3h. Other details as in Figure S1C and S1D. (B) Cell cycle distribution of parental A549 cells 48 h after treatment with siNC, siSKP2, siCDH1 or siSKP2 + siCDH1. (C) WB analysis of CDH1, SKP2, and P27 in A549 cells treated as in B. (D) Resection analysis in A549 cells depleted of SKP2 and CDH1. Other details as in Figure S1C and S1D. (E) As in

(D) for A549-CDH1^-/-^ cells. (F) Cell cycle distribution of G2-enriched A549-CDH1^-/-^ cells treated as described in (E).

**Figure S7:** (A) Cell cycle distributions and gates applied to quantitate RAD51 foci formation in EdU- and EdU+ 82-6 hTert cells.

**Figure S8:** (A) Effect of SKP2 knockdown on GC as measured using an A549 cell line engineered with a genomic integration of the DR-GFP construct (P=0.019). (B) Experiment similar to that described in (A) but for U2OS DR-GFP cells (P=0.058). (C) Cell cycle distributions of G2 enriched 82-6 hTert cells 48 h after transfection with siNC, siSKP2 or siCTIP. (D) Cell cycle distributions of G2 enriched 82-6 hTert cells 48 h after transfection with siNC, siCDH1, siSKP2 or siCDH1+siSKP2.
