# Supplementary figures and images for: SCFSKP2 regulates APC/CCDH1-mediated degradation of CTIP to adjust DNA-end resection in G2-phase
Source: Cell Death Dis. 2020 Jul 18;11(7):548. doi: 10.1038/s41419-020-02755-9 (PMC7368859; doi:10.1038/s41419-020-02755-9)

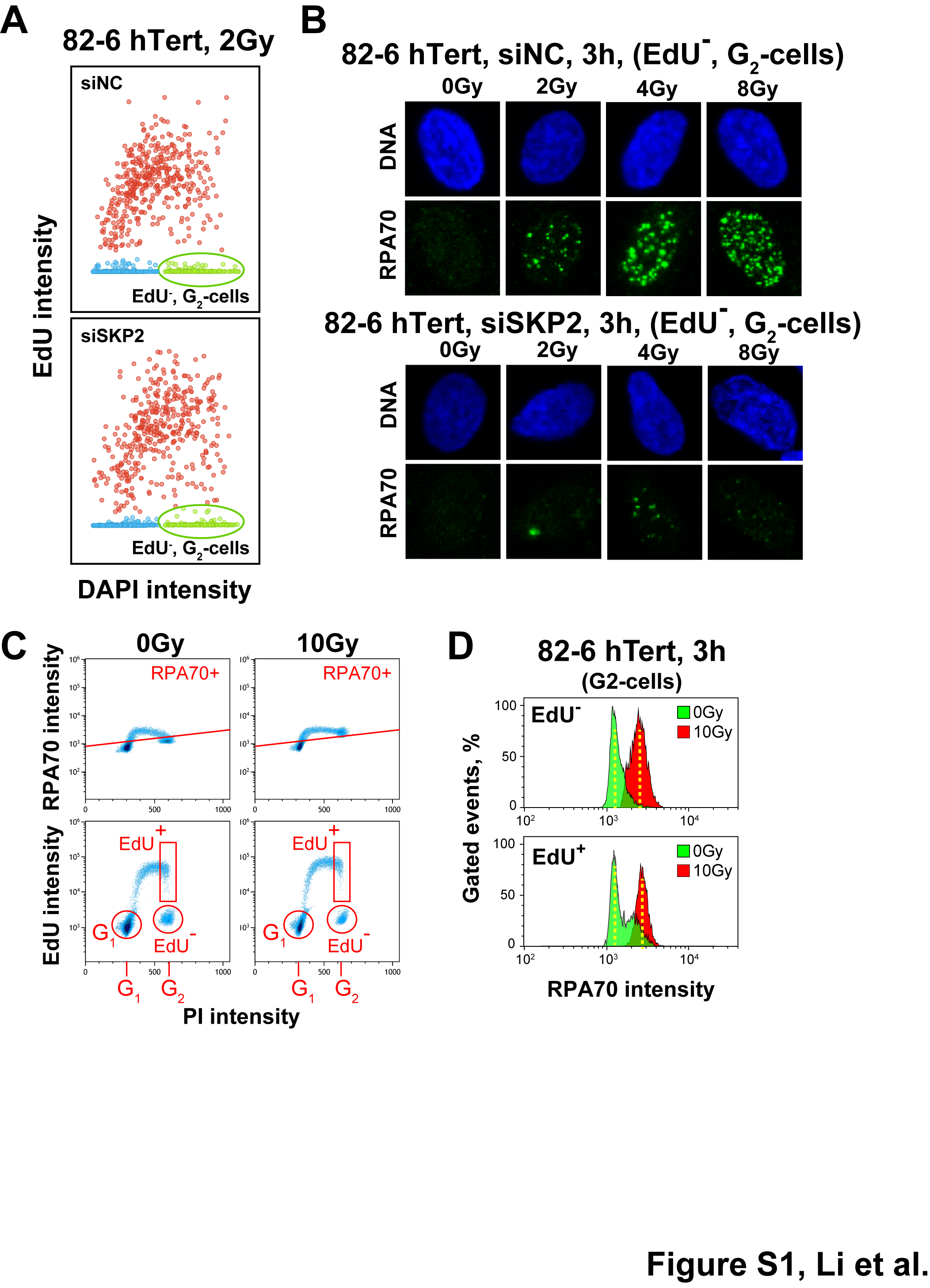

Supplement: Supplementary file 2 — Supplementary Information [file 41419_2020_2755_MOESM2_ESM.tif]

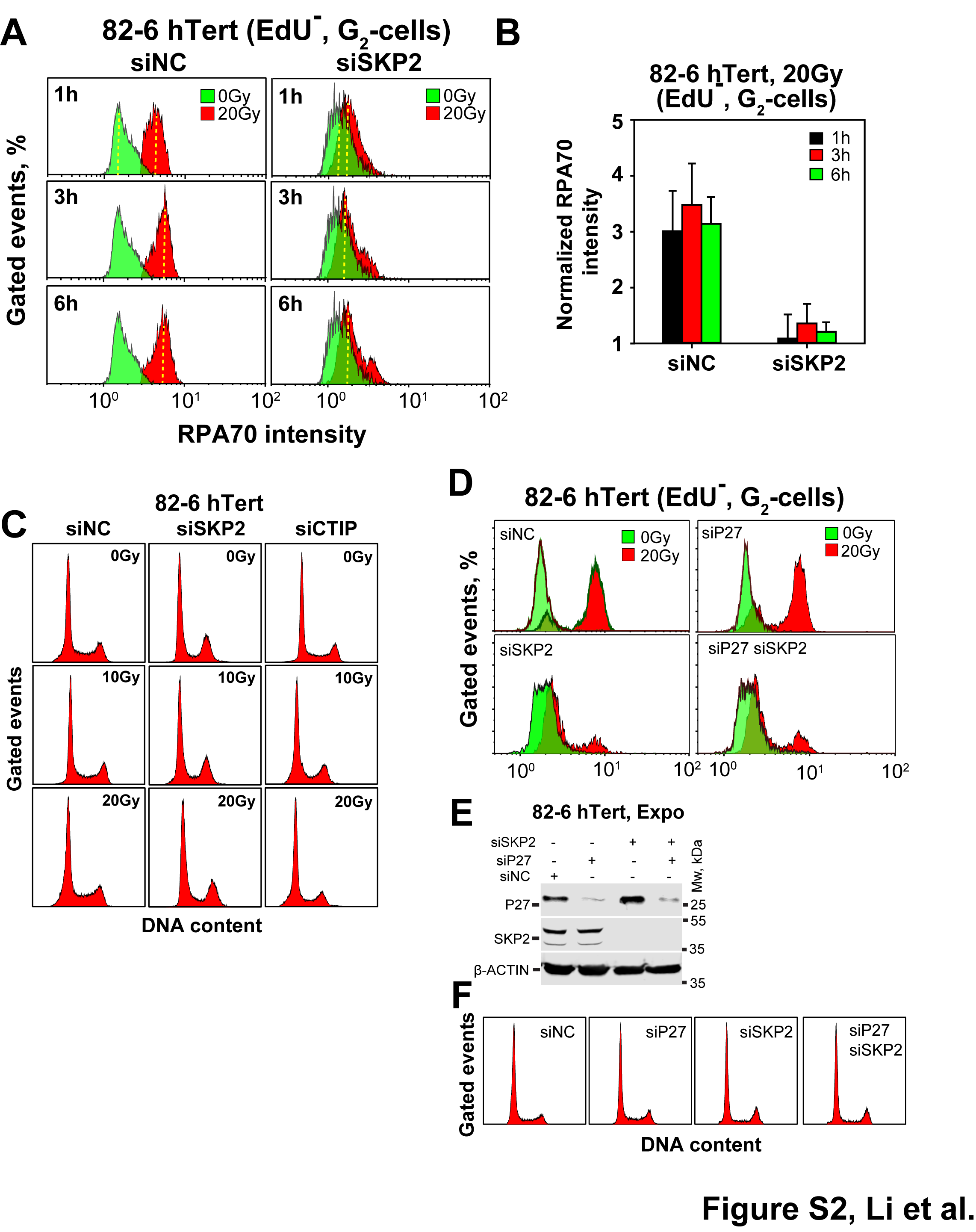

Supplement: Supplementary file 3 — Supplementary Information [file 41419_2020_2755_MOESM3_ESM.tif]

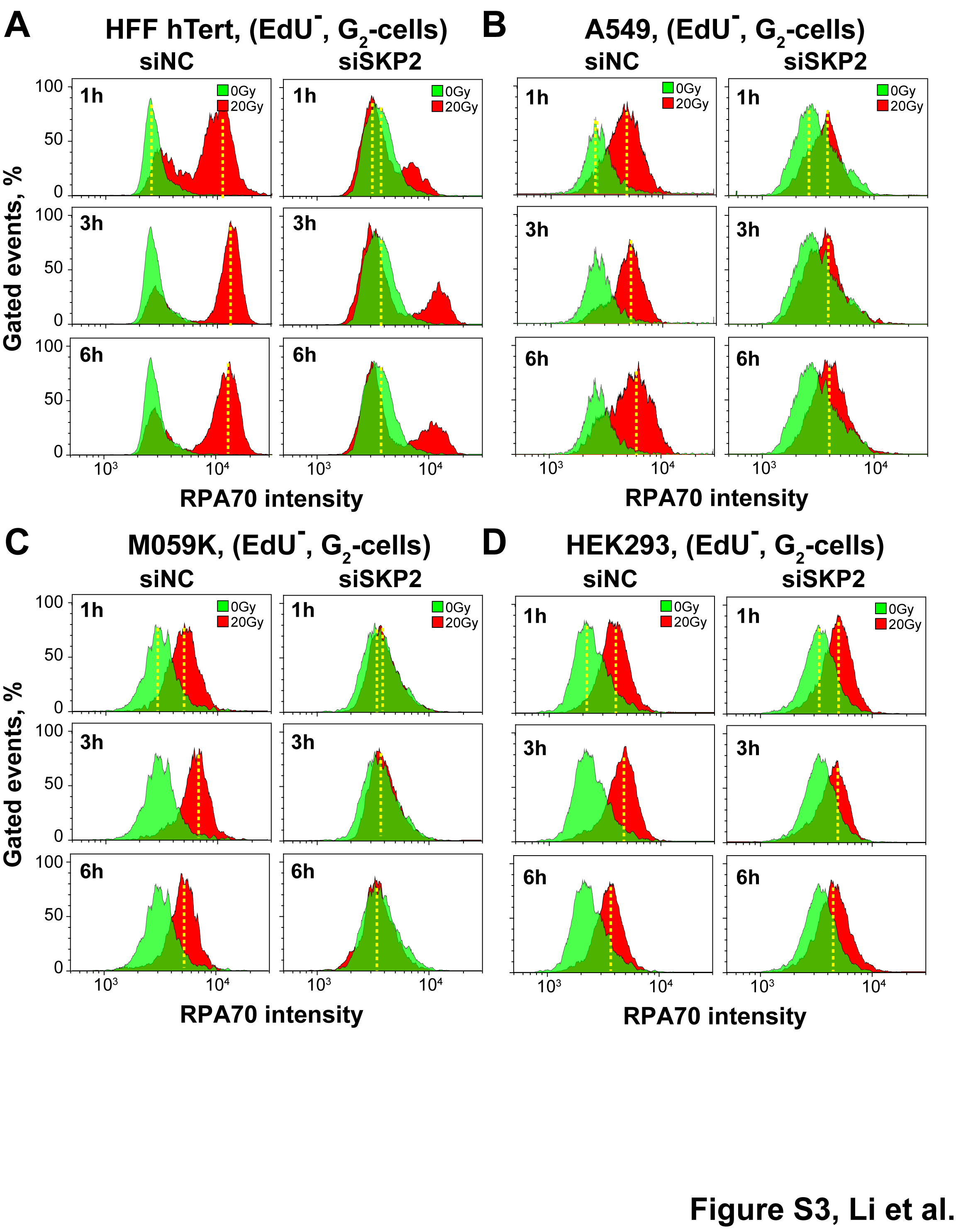

Supplement: Supplementary file 4 — Supplementary Information [file 41419_2020_2755_MOESM4_ESM.tif]

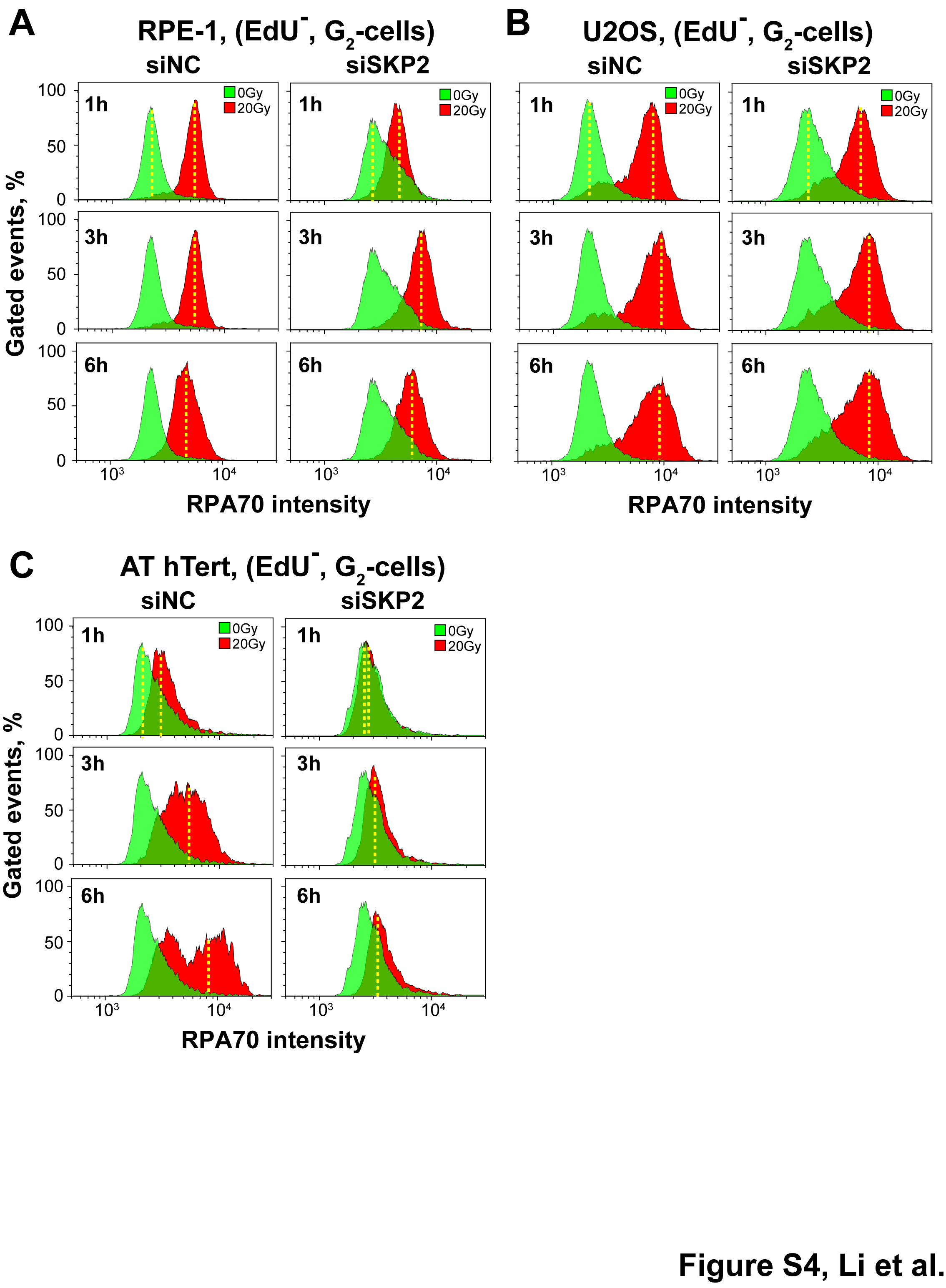

Supplement: Supplementary file 5 — Supplementary Information [file 41419_2020_2755_MOESM5_ESM.tif]

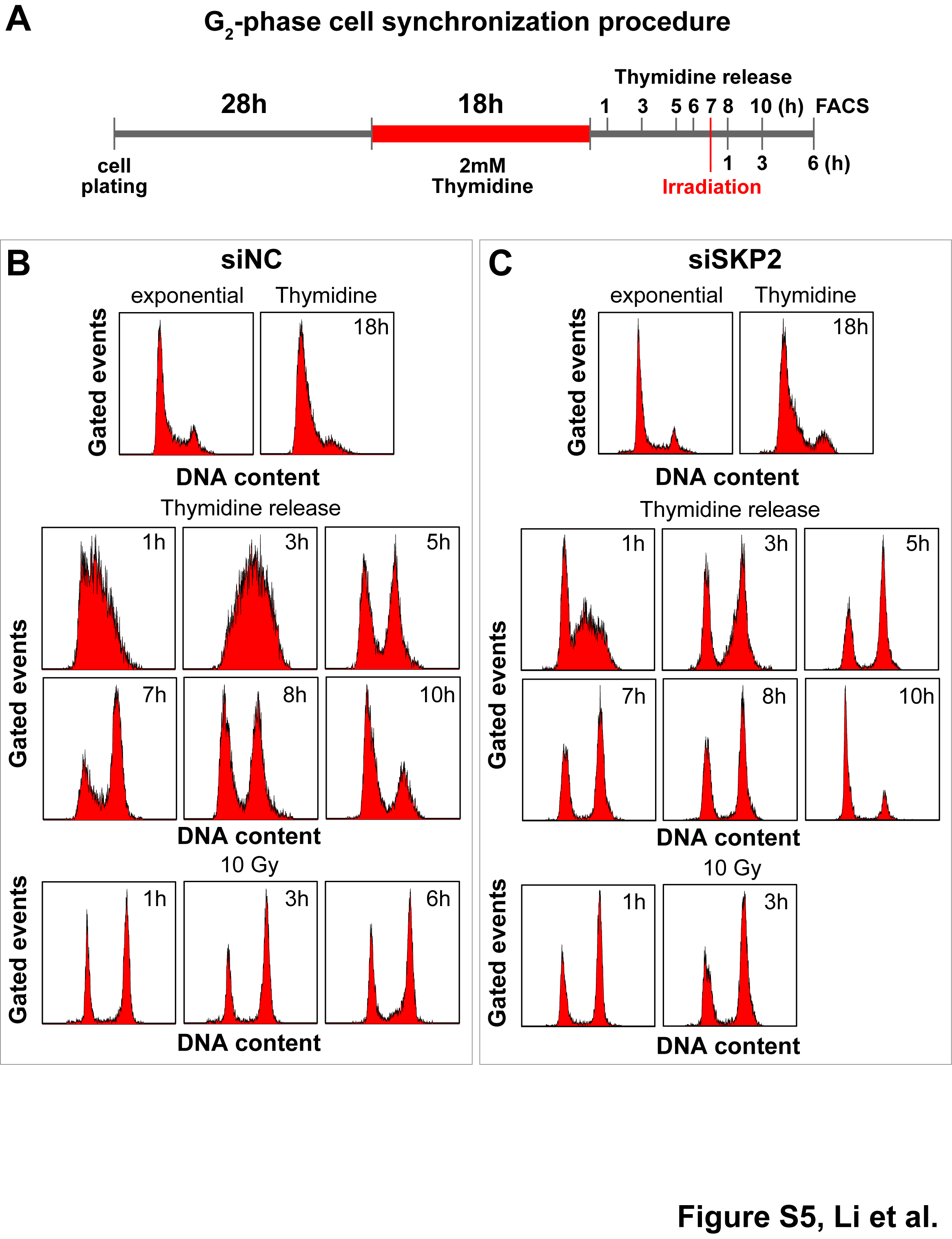

Supplement: Supplementary file 6 — Supplementary Information [file 41419_2020_2755_MOESM6_ESM.tif]

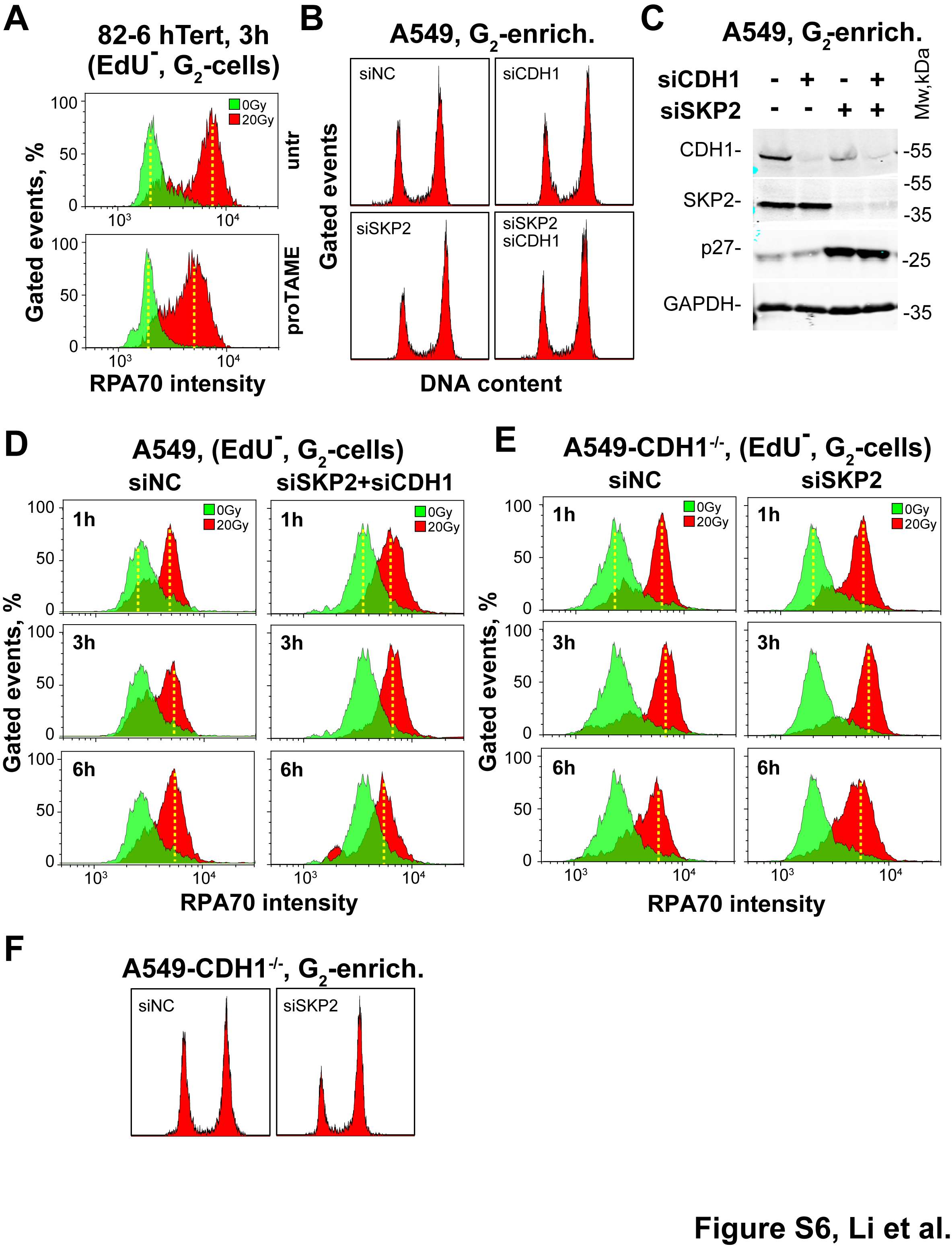

Supplement: Supplementary file 7 — Supplementary Information [file 41419_2020_2755_MOESM7_ESM.tif]

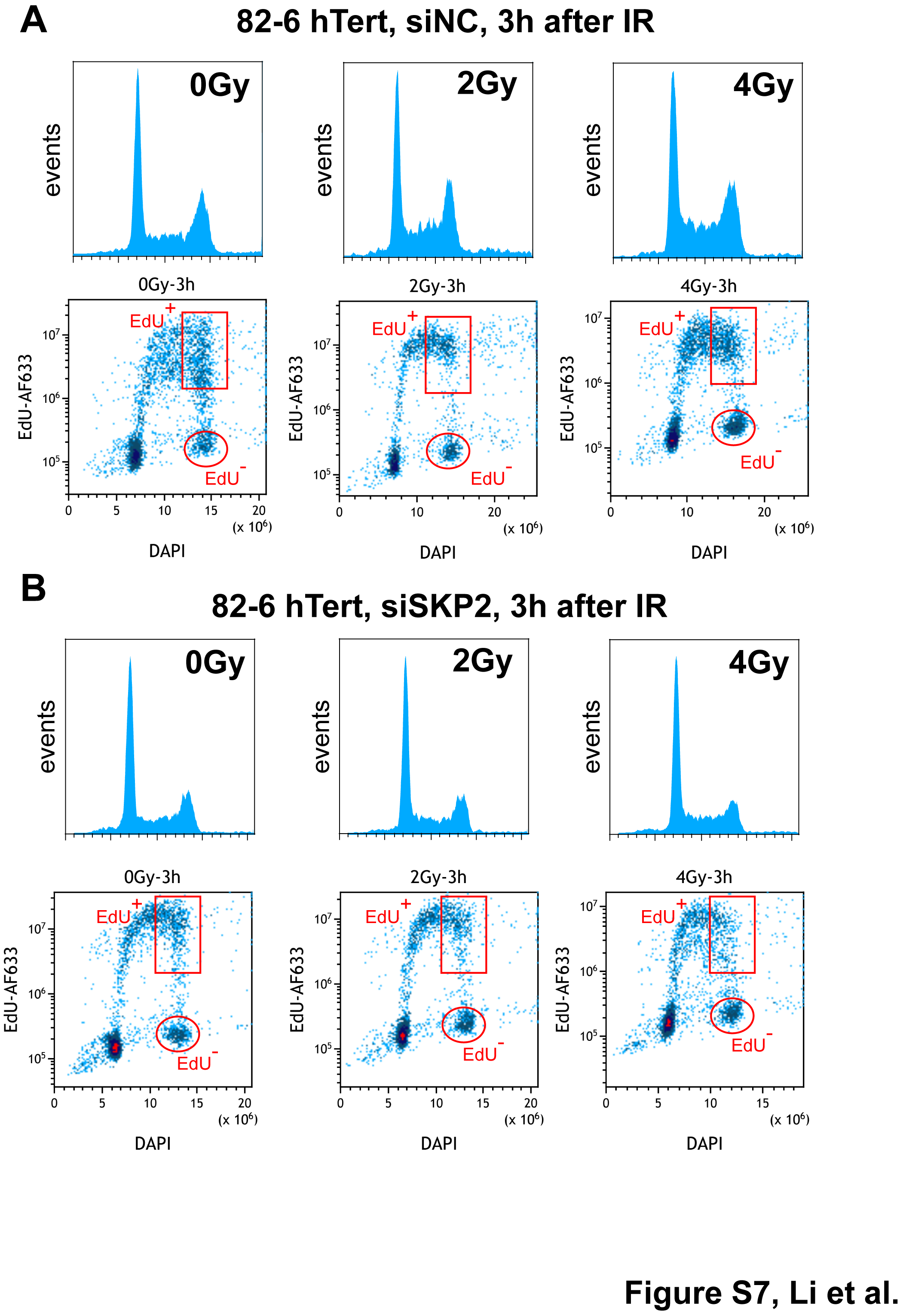

Supplement: Supplementary file 8 — Supplementary Information [file 41419_2020_2755_MOESM8_ESM.tif]

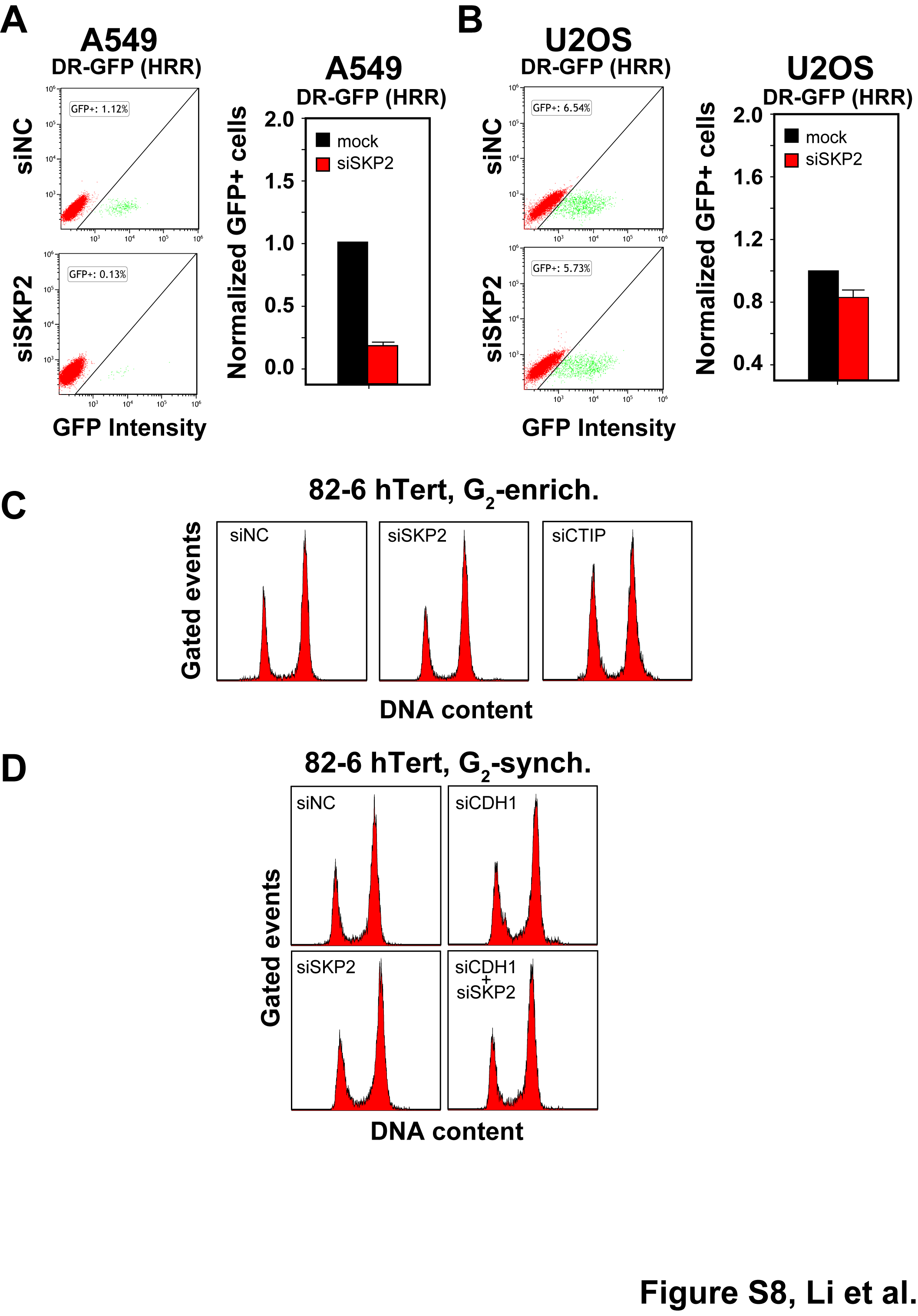

Supplement: Supplementary file 9 — Supplementary Information [file 41419_2020_2755_MOESM9_ESM.tif]
