# Supplementary material for: SCFSKP2 regulates APC/CCDH1-mediated degradation of CTIP to adjust DNA-end resection in G2-phase
Source: Cell Death Dis. 2020 Jul 18;11(7):548. doi: 10.1038/s41419-020-02755-9 (PMC7368859; doi:10.1038/s41419-020-02755-9)
Supplement: Supplementary file 10 — Supplementary Information [file 41419_2020_2755_MOESM10_ESM.docx]

**Supplementary Material and Methods**

**Antibodies**

| **Name** | **Provider** | **Designation of provider** |
| --- | --- | --- |
| Mouse monoclonal anti-RPA70B | Dr. J. Hurwitz ^1^ | N/A |
| Rabbit monoclonal anti-CTIP | Cell Signaling Technonology | Cat#9201 |
| Mouse monoclonal anti-FZR/CDH1 | Santa Cruz Biotechnology | Cat# sc-56312 |
| Mouse monoclonal anti-SKP2 | Thermo Fisher Scientific | Cat# 32-3300 |
| Mouse monoclonal anti-PLK1 | Thermo Fisher Scientific | Cat# 33-1700 |
| Mouse monoclonal anti-CCNB1 | Santa Cruz Biotechnology | Cat# sc-245 |
| Rabbit polyclonal anti-CCNA2 | Santa Cruz Biotechnology | Cat# sc-596 |
| Rabbit polyclonal anti-CCND1 | Cell Signaling Technonology | Cat# 2922 |
| Rabbit polyclonal anti-MRE11 | Novus Biologicals | Cat# NB100-142 |
| Mouse monoclonal anti-NBS1/NIBRIN | Santa Cruz Biotechnology | Cat# sc-374168 |
| Mouse monoclonal anti-RAD50 | GeneTex | Cat# GTX70228 |
| Mouse monoclonal anti-P27 | Becton Dickinson | Cat# 610242 |
| Rabbit polyclonal anti-GAPDH | Santa Cruz Biotechnology | Cat# sc-25778 |
| Mouse monoclonal anti-KU70 | GeneTex | Cat# GTX23114 |
| Mouse monoclonal anti-RPA32 | Dr. J. Hurwitz ^1^ | N/A |
| Mouse monoclonal anti-KAP1 | Abcam | Cat# ab22553 |
| Rabbit polyclonal anti KAP1-pS824 | Bethyl | Cat# A300-767A |
| Rabbit polyclonal anti-USP4 | Cell Signaling Technology | Cat#2651 |
| Rabbit polyclonal anti-KU80 | Cell Signaling Technology | Cat#2753 |
| Mouse monoclonal anti-CUL1 | Santa Cruz Biotechnology | Cat# sc-17775 |
| Alexa Fluor 488 Goat anti-Mouse IgG (H+L) | Thermo Fisher Scientific | Cat# A11001 |
| IRDye 680RD Goat anti-Mouse IgG (H+L) | LI-COR Biosciences | Cat#926-68020 |
| IRDye 800CW Goat anti-Mouse IgG (H+L) | LI-COR Biosciences | Cat#926-32210 |
| IRDye 680RD Goat anti-Rabbit IgG (H+L) | LI-COR Biosciences | Cat#926-68021 |
| IRDye 800CW Goat anti-Rabbit IgG (H+L) | LI-COR Biosciences | Cat#926-32211 |

**Oligonucleotides**

| **Name** | **Provider** | **Designation of provider** |
| --- | --- | --- |
| siRNA targeting CTIP: GCUAAAACAGGAACGAAUC | ^2^ | N/A |
| siRNA targeting Skp2: [Hs_SKP2_5 FlexiTube siRNA](https://www.qiagen.com/de/shop/rnai/flexitube-sirna/?catno=SI00287819) | QIAGEN | Cat#SI00287819 |
| siRNA targeting FZR1/CDH1: GGAUUAACGAGAAUGAGAA | ^3^ | N/A |
| siRNA targeting CDC14B: GAUGCUACAUGGUUUAUA | ^5^ | N/A |
| siRNA targeting SKP1: CGCAAGACCUUCAAUAUCA | ^4^ | N/A |
| siRNA targeting CUL1: GUUCAUAGCAGCCAGCCUG | ^6^ | N/A |
| siRNA targeting USP4:  UUAAACAGGUGGUGAGAAA | ^7^ | N/A |
| siRNA targeting P27: GGAGCAAUGCGCAGGAAUAUU | ^8^ | N/A |
| negative control siRNA: UUCUCCGAACGUGUCACGU | ^9^ | N/A |
| sgRNA targeting FZR1/CDH1:  1.GCCCTGCCTCGCCATGGACC  2.GCCACCTCAGACAACGGCAA  3.GGTGCAGGACCCGCAGACTG  4.GAGGGCCACACGGCACGCGT  5.CCCCGCCACTGCAGTCGGAG | This paper | N/A |

**Cells**

| **Name** | **Provider** | **Designation of provider** |
| --- | --- | --- |
| 82-6 hTert | [Markus Löbrich](https://www.ncbi.nlm.nih.gov/pubmed/?term=L%26%23x000f6%3Bbrich%20M%5BAuthor%5D&cauthor=true&cauthor_uid=23935532) ^10^ | N/A |
| A549: wild type | ATCC | ATCC CCL-185 |
| A549: FZR1/CDH1^-/-^ | This paper | N/A |
| A549-DR-GFP | This paper | N/A |
| U2OS | ATCC | ATCC HTB-96 |
| U2OS-DR-GFP | J. Stark ^11^ | N/A |
| RPE-1 | ATCC | ATCC CRL-4000 |
| HFF hTert | ExPASy | CVCL-VC40 |
| M059K | ATCC | ATCC CRL-2365 |
| HEK293 | ATCC | ATCC CRL-1573 |
| RPE-1 | ATCC | ATCC CRL-4000 |
| AT hTert | ExPASy | CVCL-VL09 |

**Supplementary** **References**

1. Kenny, M.K., Schlegel, U., Furneaux, H. & Hurwitz, J. The role of human single-stranded DNA binding protein and its individual subunits in simian virus 40 DNA replication. *Journal of Biological Chemistry* **265**, 7693-7700 (1990).

2. Barton, O. et al. Polo-like kinase 3 regulates CtIP during DNA double-strand break repair in G1. *Journal of Cell Biology* **206**, 877-894 (2014).

3. Lafranchi, L. et al. APC/CCdh1 controls CtIP stability during the cell cycle and in response to DNA damage. *EMBO Journal* **33**, 2860-2879 (2014).

4. Salahudeen, A.A. et al. An E3 ligase possessing an iron-responsive hemerythrin domain is a regulator of iron homeostasis. *Science* **326**, 722-6 (2009).

5. Bassermann, F. et al. The Cdc14B-Cdh1-Plk1 axis controls the G2 DNA-damage-response checkpoint. *Cell* **134**, 256-267 (2008).

6. Van Rechem, C. et al. The SKP1-Cul1-F-box and leucine-rich repeat protein 4 (SCF-FbxL4) ubiquitin ligase regulates lysine demethylase 4A (KDM4A)/Jumonji domain-containing 2A (JMJD2A) protein. *J Biol Chem* **286**, 30462-70 (2011).

7. Wijnhoven, P. et al. USP4 Auto-Deubiquitylation Promotes Homologous Recombination. *Molecular Cell* **60**, 362-373 (2015).

8. Hu, R. & Aplin, A.E. Skp2 Regulates G2/M Progression in a p53-dependent Manner. *Molecular Biology of the Cell* **19**, 4602-4610 (2008).

9. Elbashir, S.M. et al. Duplexes of 21-nucleotide RNAs mediate RNA interference in cultured mammalian cells. *Nature* **411**, 494-8 (2001).

10. Geuting, V., Reul, C. & Löbrich, M. ATM Release at Resected Double-Strand Breaks Provides Heterochromatin Reconstitution to Facilitate Homologous Recombination. *PLoS Genetics* **9**, e1003667 (2013).

11. Gunn, A., Bennardo, N., Cheng, A. & Stark, J.M. Correct End Use during End Joining of Multiple Chromosomal Double Strand Breaks Is Influenced by Repair Protein RAD50, DNA-dependent Protein Kinase DNA-PKcs, and Transcription Context. *Journal of Biological Chemistry* **286**, 42470-42482 (2011).
